# Nonreciprocal superconducting NbSe_2_ antenna

**DOI:** 10.1038/s41467-020-19459-5

**Published:** 2020-11-06

**Authors:** Enze Zhang, Xian Xu, Yi-Chao Zou, Linfeng Ai, Xiang Dong, Ce Huang, Pengliang Leng, Shanshan Liu, Yuda Zhang, Zehao Jia, Xinyue Peng, Minhao Zhao, Yunkun Yang, Zihan Li, Hangwen Guo, Sarah J. Haigh, Naoto Nagaosa, Jian Shen, Faxian Xiu

**Affiliations:** 1 State Key Laboratory of Surface Physics and Department of Physics, Fudan University, Shanghai, 200433 China; 2 Institute for Nanoelectronic Devices and Quantum Computing, Fudan University, Shanghai, 200433 China; 3 School of Physics Science and Engineering, Tongji University, Shanghai, 200092 China; 4 Department of Materials, University of Manchester, Manchester, M13 9PL UK; 5 Department of Applied Physics, The University of Tokyo, Tokyo, 113-8656 Japan; 6 RIKEN Center for Emergent Matter Science (CEMS), Saitama, 351-0198 Japan; 7 Collaborative Innovation Center of Advanced Microstructures, Nanjing University, Nanjing, 210093 China; 8 Shanghai Research Center for Quantum Sciences, Shanghai, 201315 China

**Keywords:** Superconducting devices, Superconducting properties and materials

## Abstract

The rise of two-dimensional (2D) crystalline superconductors has opened a new frontier of investigating unconventional quantum phenomena in low dimensions. However, despite the enormous advances achieved towards understanding the underlying physics, practical device applications like sensors and detectors using 2D superconductors are still lacking. Here, we demonstrate nonreciprocal antenna devices based on atomically thin NbSe_2_. Reversible nonreciprocal charge transport is unveiled in 2D NbSe_2_ through multi-reversal antisymmetric second harmonic magnetoresistance isotherms. Based on this nonreciprocity, our NbSe_2_ antenna devices exhibit a reversible nonreciprocal sensitivity to externally alternating current (AC) electromagnetic waves, which is attributed to the vortex flow in asymmetric pinning potentials driven by the AC driving force. More importantly, a successful control of the nonreciprocal sensitivity of the antenna devices has been achieved by applying electromagnetic waves with different frequencies and amplitudes. The device’s response increases with increasing electromagnetic wave amplitude and exhibits prominent broadband sensing from 5 to 900 MHz.

## Abstract (web summary)

Here, the authors observe reversible nonreciprocal charge transport in two-dimensional NbSe_2_, and demonstrate antenna devices exhibiting strong sensitivity to driving AC electromagnetic waves in the superconducting regime.

## Introduction

Crystal symmetry plays an essential role in condensed matter physics. Breaking inversion symmetry in materials profoundly changes the electronic ground states of materials and thus brings about many novel physical properties and functionalities, such as nonlinear Hall effect in Weyl semimetals^1,2^, chiral optical responses in semiconductors^3,4^ and ferroelectricity^5^. One particular example is the nonreciprocal charge transport in systems with both broken inversion and time-reversal symmetries^6^, where the electrical resistivity of a conductor is expected to vary depending on the current and magnetic field direction. Experimentally, nonreciprocal charge transport has been demonstrated in Bi helix^7^, chiral magnet^8,9^, Rashba semiconductor^10^, LaAlO_3_/SrTiO_3_ oxide interface^11^ and in various superconducting systems. These include superconducting non-centrosymmetric gated-MoS_2_^12^, Bi_2_Te_3_/FeTe heterostructures^13^ and MoGe/Y_3_Fe_5_O_12_ bilayers^14^, where the nonreciprocal response is markedly enhanced by several orders of magnitude compared to non-superconducting systems due to the large energy scale difference between the Fermi energy and the superconducting gap^12,15^. Apart from being a powerful tool to study the interplay between superconductivity and chirality in non-centrosymmetric superconductors^6,16^, nonreciprocal charge transport also promises great potential in superconducting device applications such as vortex diodes^17^ and flux lenses^18^, both of which are in great demand for future electrical circuits.

Atomically thin NbSe_2_ is an emerging non-centrosymmetric superconductor possessing unique intrinsic Ising-type spin-orbit coupling, in which the electron spin is locked to the out-of-plane direction^19,20^. Accordingly, many exotic superconducting characteristics arise, for example, extremely large upper critical fields exceeding the Pauli limit^19,21,22^ and an unusual continuous paramagnetic-limited superconductor-normal metal transition^20^. Meanwhile, as the thickness is reduced to the atomic scale where the fluctuation and disorder begin to play roles, 2D NbSe_2_ becomes very sensitive to environmental perturbations^23^. Based on the aforementioned good merits, 2D NbSe_2_ provides an ideal platform for exploring new mechanisms of nonreciprocal charge transport in non-centrosymmetric superconductors^15,24^ and further device applications^25^. In particular, compared with conventional diodes that utilize a built-in electric field in semiconductor junctions, rectifiers based on atomically thin superconductors using the intrinsic electronic properties of quantum crystals pave the way towards the realization of high-frequency sensors and detectors for next-generation wireless networks^26^. However, practical sensing devices based on layered superconductors are still lacking.

Here we report the observation of nonreciprocal charge transport in atomically thin 2D NbSe_2_ and the demonstration of successful manipulation of nonreciprocal sensitivity in atomically thin NbSe_2_ antenna devices. The second harmonic magnetoresistance of few-layer NbSe_2_ exhibits multiple antisymmetric reversals when the temperature is below the superconducting transition temperature *T*_*C*_, manifesting itself as a feature of reversible nonreciprocal charge transport due to the broken inversion symmetry. Utilizing the reversible nonreciprocal charge transport in NbSe_2_, we have built superconducting antenna devices that exhibit a strong reversible nonreciprocal sensitivity to the applied alternating current (AC) electromagnetic waves. Furthermore, we find that the response of the antenna increases monotonically with the increasing amplitude of the electromagnetic waves and that the devices show prominent broadband sensing from 5 to 900 MHz. Our research not only demonstrates the exotic physics in 2D NbSe_2_ but also establishes it to be a promising platform for radio-frequency energy-harvesting, sensing, and identification applications.

## Results

### Nonreciprocal charge transport in 2D NbSe_2_

**Fig. 1:**
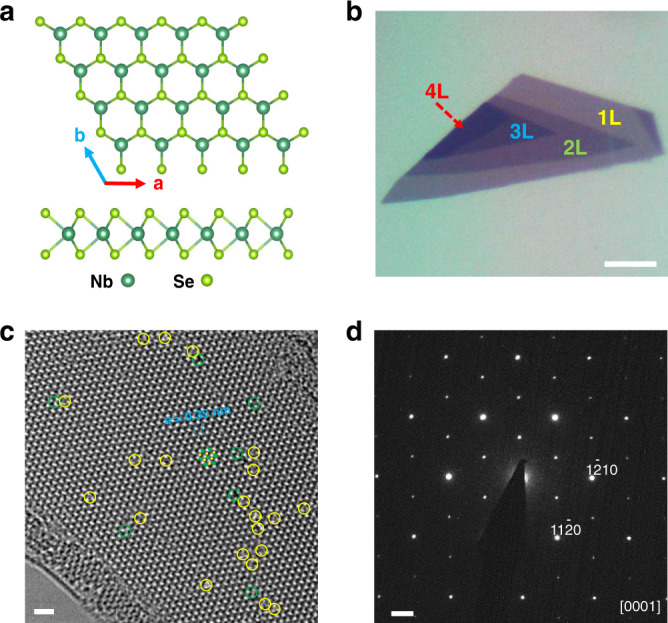
Crystal structure and characterization of NbSe_2_. **a**. Top and side views of NbSe_2_ crystal. Nb atoms (shown dark green) are sandwiched between two layers of Se (shown light green) atoms. Monolayer NbSe_2_ exhibits spatial inversion symmetry breaking because the Nb and Se sites are not equivalent. **b**. Optical image of a few-layer flake of NbSe_2_ cleaved on a SiO_2_/Si substrate with a spin-coated PMMA/MMA polymer on it. The number of layers is labeled. Scale bar: 5 μm. **c**. High-resolution TEM image taken from a NbSe_2_ flake suspended on a TEM grid, in which the Nb and Se atomic columns are marked by green and yellow dots. And the selected point defects located at Nb and Se sites are highlighted by green and yellow circles, respectively. Scale bar: 1 nm. **d**. SAED taken from the NbSe_2_ flake along [0001] zone axis. Scale bar: 2 nm^−1^.

Figure 1a shows the typical crystal structure of NbSe_2_. It has a hexagonal lattice structure within the *a-b* plane and crystalizes with the P63/mmc space group^27^. Monolayer NbSe_2_ consists of a sublayer of Nb atoms sandwiched between two sublayers of Se atoms in the trigonal prismatic structure^19,28^. Spatial inversion symmetry is broken in monolayer NbSe_2_ because the Nb and Se sites are not equivalent. Various thicknesses of atomically thin NbSe_2_ were obtained via exfoliation of bulk crystals onto SiO_2_/Si substrates (see Methods for details). Figure 1b displays an optical image of an exfoliated few-layer NbSe_2_ flake where the number of layers is marked. Figure 1c shows an atomic-resolution transmission electron microscopy (TEM) image taken from an atomically thin exfoliated NbSe_2_ flake. In perfect regions of the crystal, the atomic arrangement agrees well with the expected 2H crystal structure of NbSe_2_^29^. However, Fig. 1c shows that some point defects (highlighted by circles) can be observed locally, even in high-quality exfoliated materials. This kind of point defects can act as asymmetric pinning potentials in superconducting regimes^30–32^ which we will discuss later. Figure 1d displays the selected-area electron diffraction (SAED) pattern, confirming that the exfoliated flake is a single crystal and its dominant surface is {0001}.

As is typical for systems with broken inversion symmetry, nonreciprocal charge transport, so-called magneto-chiral anisotropy, will appear when time-reversal symmetry is broken by an external magnetic field^24,33^. Under such circumstances, the electrical resistance of the device will depend on the current direction and can be phenomenologically described as^7,12,24,34^

1 $$R = R_0(1 + \gamma IB).$$

**Fig. 2:**
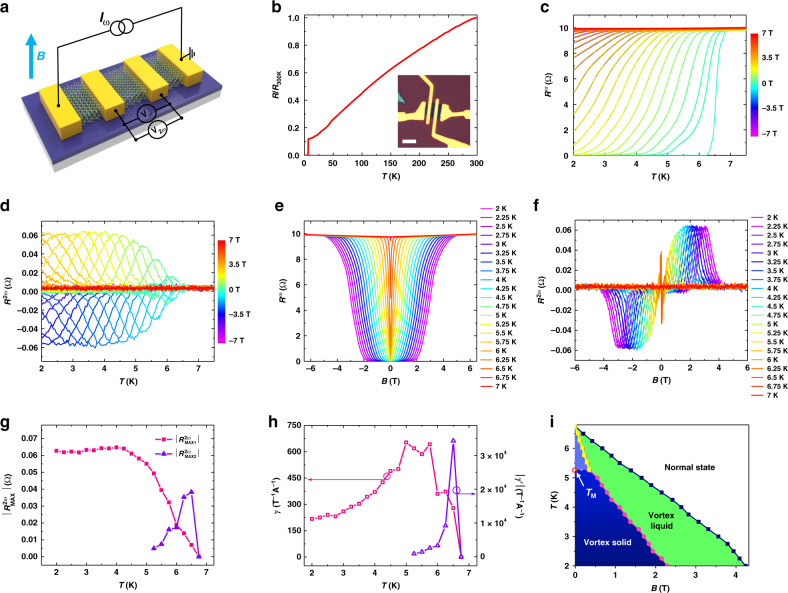
Nonreciprocal charge transport in atomically thin NbSe_2_. **a**, Sketch of the four-terminal device based on atomically thin NbSe_2_. The magnetic field is perpendicularly to the substrate plane. **b**, The temperature dependence of the normalized resistance of the NbSe_2_ device. Inset, an optical image of the four-terminal NbSe_2_ device with a thickness of five layers. Scale bar, 5 μm. **c**, **d**, Temperature-dependent *R*^*ω*^ and *R*^*2ω*^ of the device under positive and negative magnetic fields applied perpendicularly to the substrate, where *R*^*ω*^*(B)-T* and *R*^*ω*^*(-B)-T* curves overlap each other while *R*^*2ω*^*(B)-T* and *R*^*2ω*^*(-B)-T* curves are symmetric with respect to the *x*-axis (*I*_*0*_ = 25 μA and *f* = 17.1 Hz). **e**, Magnetoresistance isotherm of the device with the temperature changing from 2 to 7 K, which are symmetric with respect to the *y*-axis. **f**, *R*^*2ω*^*-B* curves of the device at temperatures of 2 to 7 K, showing antisymmetric behavior at *T* < 7 K, which is consistent with the first harmonic signal in (**e**). **g**, The extracted maximum value of *R*^*2ω*^*-B* curves $\left| {R_{MAX}^{2\omega }} \right|$ as a function of temperature. The pink squares $\left| {R_{MAX1}^{2\omega }} \right|$ stands for the peak values in the larger magnetic field regime, the purple triangles $\left| {R_{MAX2}^{2\omega }} \right|$ stands for the peak values in the small magnetic field regime at B ≥ 5.25 K. **h**, Calculated temperature-dependent *γ* and *│γ*′│, where $\gamma = \frac{{\sqrt 2 \left| {R_{MAX1}^{2\omega }} \right|}}{{BR^\omega I_0}}$ and $\gamma ^{\prime} = \frac{{\sqrt 2 \left| {R_{MAX2}^{2\omega }} \right|}}{{BR^\omega I_0}}$, respectively. *γ* and *γ*′ have an opposite sign due to the opposite nonreciprocity. **i**, Temperature-magnetic field phase diagram of the NbSe_2_ device. The dark pink dots stand for crossover between the vortex solid (glass) state and vortex liquid state at which *R*^*2ω*^ goes to zero. The dark blue dots show the crossing point of the vortex liquid state and normal state. *R*^*2ω*^ goes to zero when further increasing the magnetic field. The yellow dots are the crossing point where *R*^*2ω*^ changes sign, defining the boundary between the activated pinned vortex states (light blue area) and vortex liquid state at *T*_M_ ≥ 5.25 K. Here *T*_M_ is the melting temperature above which the vortex solid melts.

Here, *R*_0_ represents the resistance at zero magnetic field, *I* is the electrical current, *B* is the external magnetic field and *γ* is a coefficient representing the strength of the magneto-chiral anisotropy effect(see Supplementary Note 1 for detail). Based on Eq. (1), we have carried out nonreciprocal charge transport experiments using a typical device structure shown in Fig. 2a with the optical image in Fig. 2b inset. The first and second harmonic magnetoresistances were measured simultaneously. The temperature-dependent normalized resistance *R/R*_*300 K*_ of a five-layer device is shown in Fig. 2b. The sample exhibits a metallic behavior upon cooling and becomes superconducting at *T*_*C*_ = 6.5 K (*T*_*C*_ is defined as the temperature corresponding to 50% of the resistance above the superconducting transition *R*_*N*_). Figure 2c illustrates the temperature-dependent first harmonic resistance *R*^*ω*^ with a perpendicular magnetic field from −7 to 7 T, in which each *R*^*ω*^*(B)-T* curve overlaps with *R*^*ω*^*(-B)-T* curve. Interestingly, for the temperature-dependent *R*^*2ω*^ as depicted in Fig. 2d, the *R*^*2ω*^*(B)-T* and *R*^*2ω*^*(-B)-T* curves are symmetric with respect to the *x*-axis. These phenomena are also consistent with the behaviors of first harmonic and second harmonic magnetoresistance isotherms shown in Fig. 2e, f, where *R*^*ω*^*-B* and *R*^*2ω*^*-B* curves are respectively symmetric and antisymmetric with respect to the *y*-axis. The antisymmetric feature of *R*^*2ω*^*-B* curve is consistent with Eq. (1) which unambiguously suggests the existence of the magneto-chiral anisotropy in 2D NbSe_2_^12,15,24^.

Furthermore, the *R*^*2ω*^*-B* curve shows one pair of peaks at low temperatures in the first and third quadrant (*T* ≤ 5 K) and as the temperature increases further (*T* > 5 K), another pair of peaks emerge in the second and fourth quadrant (also see Supplementary Fig. 2). The extracted peak values of the *R*^*2ω*^*-B* curve $\left| {R_{MAX1}^{2\omega }} \right|$ and $\left| {R_{MAX2}^{2\omega }} \right|$ are shown in Fig. 2g, demonstrating that both $\left| {R_{MAX1}^{2\omega }} \right|$ and $\left| {R_{MAX2}^{2\omega }} \right|$ are greatly enhanced at *T* < *T*_*C*_ and $\left| {R_{MAX1}^{2\omega }} \right|$ saturates at *T* ≤ 4.5 K. The deduced *γ* and *γ´* values as a function of temperature are shown in Fig. 2h, here γ and γ´ are defined as $\gamma = \frac{{\sqrt 2 \left| {R_{MAX1}^{2\omega }} \right|}}{{BR^\omega I_0}}$ and $\gamma ^{\prime} = \frac{{\sqrt 2 \left| {R_{MAX2}^{2\omega }} \right|}}{{BR^\omega I_0}}$, respectively (the corresponding values of *B* and *R*^*ω*^ were used to calculate *γ* and *γ*′). The maximum of *γ* and *γ*′ are 6.53 × 10^2^ T^−1^A^−1^ and 3.43 × 10^4^ T^−1^A^−1^, respectively. Both of them are higher than those reported in other non-superconducting systems such as Bi helix (*γ* ∼ 10^−3^ A^−1^ T^−1^)^34^, chiral organic materials (*γ* ∼ 10^−2^ A^−1^ T^−1^)^33^, BiTeBr (*γ* ∼ 1 A^−1^ T^−1^)^10^ and LaAlO_3_/SrTiO_3_ oxide interface^11^ (*γ* ∼ 10^2^ A^−1^ T^−1^). The normalized coefficient value which defined as $\gamma _N = \gamma A$ and $\gamma ^{\prime}_N = \gamma ^{\prime}A$ (*A* here is the cross-sectional area of device) are 1.44 × 10^−11^ T^−1^A^−1^m^2^ and 7.55 × 10^−10^ T^−1^A^−1^m^2^, respectively. Both are also higher than that observed in LaAlO_3_/SrTiO_3_ oxide interface^11^ (∼1.17 × 10^−11^ T^−1^A^−1^m^2^). The large enhancement of the nonreciprocity below the superconducting transition temperature is due to the reduction of the energy denominator from the Fermi energy (∼100 meV) to the superconducting gap (∼1 meV)^12,13,15^. Note that *R*^*2ω*^ appears only in the resistive state (because the vortex flow in 2D NbSe_2_ causes dissipation, see Supplementary Note 2 for details). In other words, *R*^*2ω*^ is nonzero only when NbSe_2_ is in the vortex flow regime, signaling the close relationship between *R*^*2ω*^ and the vortex motion. Recent theory has revealed that nonreciprocal charge transport occurs in non-centrosymmetric superconductors when vortices driven by the external charge current move among the asymmetric pinning potentials in the vortex flow regime^15^. In 2D NbSe_2_, the asymmetric pinning potentials naturally appear as a consequence of disorder^15^ such as defects^30–32^ in 2D crystals with inversion symmetry breaking, as shown in Fig. 1c. Accordingly, we attribute the emergence of another pair of *R*^*2ω*^ peaks at *T* > 5 K to the melting of the vortex solid (glass) state into the activated pinned vortex states at relatively high temperatures as shown in Fig. 2i. In the low-temperature regime (*T* ≤ 5 K), as the magnetic field increases, NbSe_2_ transforms from the non-resistive vortex solid state into the resistive vortex liquid state, in which *R*^*2ω*^ reaches its maximum value. Further increasing the magnetic field will quench the NbSe_2_ into the normal state, giving rise to a non-detectable *R*^*2ω*^. While in the high temperate regime (*T* ≥ T_M_ = 5.25 K), the vortex solid state will melt into the resistive activated pinned vortex states^35^ where *R*^*2ω*^ also appears. Then the system undergoes a transition from activated pinned vortex states to vortex liquid states as the magnetic field increases. Note that the vortex in activated pinned vortex states will be thermally activated and will jump^36^ between asymmetric pinning barriers, which is different from that in vortex liquid states where the vortex moves more freely. We then infer that the nonreciprocity of the vortex motion in activated pinned vortex states and vortex liquid states are opposite, leading to the two pairs of antisymmetric peaks in the high-temperature regime (also see Supplementary Note 2).

**Fig. 3:**
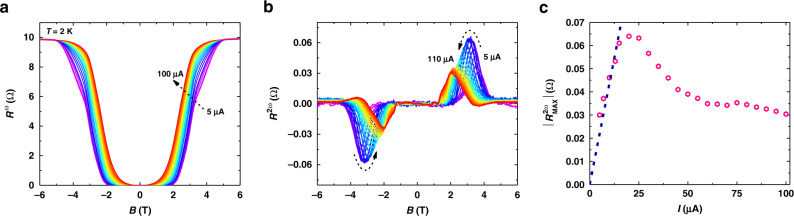
Current-dependent nonreciprocal charge transport in atomically thin NbSe_2_. **a**, Current-dependent first harmonic signal of the five-layer NbSe_2_ device at *T* = 2 K. In the small current regime (*I*_*0*_ ≤ 15 μA), the R^*ω*^-B curves are nearly unchanged with the increase of *I*_*0*_, suggesting a negligible change of the superconducting properties due to the increasing current. While in the larger current regime (*I*_*0*_ ≥ 20 μA), prominent expansion of the nonzero region in the *R*^*ω*^*-B* curves emerges, indicating a non-negligible breaking of superconducting states by increasing *I*_*0*_. **b**, Corresponding current-dependent second harmonic signal of NbSe_2_ at *T* = 2 K. **c**, Deduced maximum value $\left| {R_{MAX}^{2\omega }} \right|$ as a function of applied current, showing a linear increase at small current bias (*I*_*0*_ ≤ 15 μA) and a decreasing behavior as *I*_*0*_ increases further (*I*_*0*_ ≥ 20 μA).

Next, we try to measure the second harmonic magnetoresistance of NbSe_2_ at various applied AC currents with different amplitudes *I*_*0*_ (*I*_*0*_ is the effective value of the AC current, see Methods). Figure 3a depicts the first harmonic magnetoresistance *R*^*ω*^ of the device measured at *T* = 2 K with *I*_*0*_ changing from 5 to 100 μA. In the small current regime (*I*_*0*_ ≤ 15 μA), the *R*^*ω*^*-B* curves almost overlap with each other, indicating a negligible effect of the applied current on the superconducting states of NbSe_2_. While in the larger current regime (*I*_*0*_ ≥ 20 μA), the nonzero region (*0* < *R*^*ω*^ < *R*_*N*_) of the *R*^*ω*^*-B* curves expands as the current further increases. In other words, the larger the current is, the easier the magnetic field will bring the system into resistive states. Correspondingly, the second harmonic magnetoresistance firstly increases then decreases as *I*_*0*_ increases as shown in Fig. 3b. We extract the maximum value of $R_{MAX}^{2\omega }$ versus *I*_*0*_ in Fig. 3c. In the small current regime (*I*_*0*_ ≤ 15 μA), the maximum value of $R_{MAX}^{2\omega }$ increases linearly with the increase of *I*_*0*_, consistent with Eq. (1). As *I*_*0*_ further increases (*I*_*0*_ ≥ 20 μA), the rectification effect of vortex motion will be decreased by the relative weakening of the pinning potentials^30,37,38^. Also, the quenching of superconductivity in NbSe_2_ can no longer be neglected. As a result, $R_{MAX}^{2\omega }$ decreases as *I*_*0*_ increases at *I*_*0*_ ≥ 20 μA.

### Reversible nonreciprocal DC sensitivity in atomically thin NbSe_2_ antenna

**Fig. 4:**
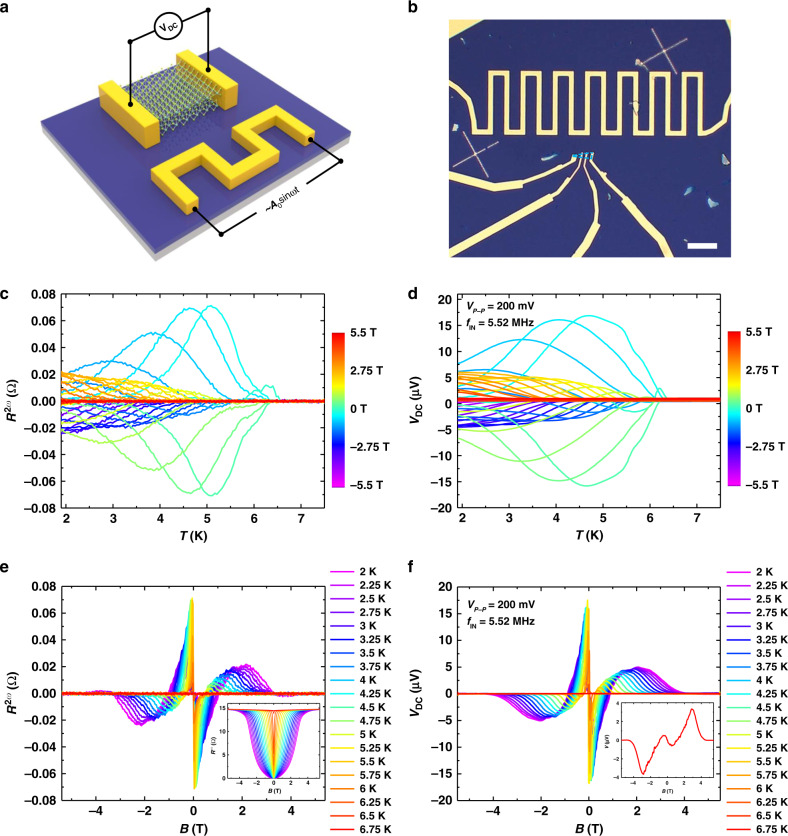
Nonreciprocal sensitivity in atomically thin NbSe_2_ antenna. **a**, Schematic structure of the NbSe_2_ antenna device for the measurement of DC signal. The nanovoltage meter was directly connected to NbSe_2_. Sine wave AC signals were applied to the fabricated resistor. **b**, Optical image of the antenna device, scale bar, 20 μm. The NbSe_2_ thickness is three layers. **c**, *R*^*2ω*^*-T* of the device under negative and positive applied magnetic fields perpendicular to the substrate (with no AC signal applied on the resistor). **d**, *V*_*DC*_*-B* of the device with sine wave AC signal (*V*_*P-P*_ = 200 mV, *f*_*IN*_ = 5.52 MHz) applied on the resistor. **e**, *R*^*2ω*^*-B* curves of the device with the temperature changing from 2 to 6.75 K. Inset, the first harmonic signal of the device measured simultaneously with the second harmonic signal. Both the first and second harmonic signals were measured under no AC signal applied to the resistor. **f**, DC signal *V*_*DC*_*-B* of the device with the temperature changing from 2 to 6.75 K with sine wave AC signal (*V*_*P-P*_ = 200 mV, *f*_*IN*_ = 5.52 MHz) applied to the resistor, showing similar antisymmetric behavior to the *R*^*2ω*^*-B* curves. Inset, DC signal of the device at *T* = 2 K with no AC signal applied to the resistor. The antisymmetric signal comes from environmental fluctuations.

Having understood the nonreciprocal charge transport in 2D NbSe_2,_ we next explore its direct current (DC) sensitivity and the relationship with nonreciprocal charge transport. We first built a NbSe_2_ antenna device in order to investigate whether or not it can respond to externally applied electromagnetic waves. The device structure is illustrated schematically in Fig. 4a with the corresponding optical image in Fig. 4b. Here an AC electromagnetic wave is applied to the resistor fabricated on the same substrate. The resistance of the resistor is intentionally designed to be ∼50 Ω so as to give an impedance matching the AC signal. Figure 4c shows the *R*^*2ω*^*(B)-T* curve of a three-layer NbSe_2_ device. The *R*^*2ω*^*(B)-T* and *R*^*2ω*^*(-B)-T* curves are symmetric with respect to the *x*-axis, consistent with the previous five-layer device in Fig. 2d. Figure 4d is the DC response of the device with the AC signal applied across the resistor under various magnetic fields (*V*_*P-P*_ = 200 mV, *f*_*IN*_ = 5.52 MHz, see Supplementary Figs. 6, 7 for additional data). The device gives a prominent DC response as the temperature drops below *T*_*C*._ Surprisingly, the *V*_*DC*_*(B)-T* and *V*_*DC*_*(-B)-T* curves are symmetric with respect to the *x*-axis, same as the *R*^*2ω*^*(B)-T* curve, suggesting a close relationship between the second harmonic signal and the DC response. As shown in Fig. 4e, f, the similarity of *R*^*2ω*^ and *V*_*DC*_ can also be observed in second harmonic magnetoresistance and *V*_*DC*_ isotherms with the temperature varying from 2 to 6.75 K. Note that the device also gives an antisymmetric DC response to the environmental fluctuation when there is no AC signal applied to the resistor (Fig. 4f inset and Supplementary Fig. 7b, the environmental fluctuations are mainly a few MHz electromagnetic waves in a cryostat^14,23^).

The similarity of *R*^*2ω*^ and *V*_*DC*_ can be explained using Eq. (1) which describes the nonreciprocal charge transport in 2D NbSe_2_ due to the vortex in asymmetric pinning potentials. If we apply an AC excitation current of $I = \sqrt 2 I_0\sin \omega t$ to the device, then the voltage of the device can be expressed as^13,15,39^

2 $$V = \sqrt 2 R_0I_0\,{\mathrm{sin}}\,\omega t + \gamma BR_0I_0^2\,{\mathrm{sin}}\left( {2\omega t - \frac{\pi }{2}} \right) + \gamma BR_0I_0^2,$$

where the first term is the first harmonic term, the second term is the second harmonic term and the third term is the DC term (see Supplementary Note 1 for a detailed derivation of the equation). Equation (2) above suggests that if we apply an AC current to the device with inversion symmetry breaking, a DC current will be generated. Then the device can be viewed as a *p-n* junction or rectifier where their asymmetry can convert an AC current passing through the device into a DC current^14^. The difference is that the device here has both an AC and DC current. In Eq. (2), the second term and the third DC term carry the same $\gamma B$ term, indicating that the second harmonic signal and the DC response should have the same relationship with the applied magnetic field. This explains the phenomena that we see in our experiments; that the *V*_*DC*_ curves of the device have a similar shape as the *R*^*2ω*^ curves. Furthermore, from the point of view of vortex motion, the asymmetric pinning potential in superconducting NbSe_2_ exerts a counterforce with different magnitudes to each direction of the AC driving force^40,41^ (introduced by the electromagnetic waves radiated onto NbSe_2_). As a result, the vortices acquire a net velocity, generating the DC voltage^17,42^ (see Supplementary Note 2). Also, because the trilayer NbSe_2_ here exhibits a much lower melting temperature than the five-layer device above, there is no vortex solid state for the device when *T* ≥ 2 K (see Supplementary Fig. 3). Consequently, the NbSe_2_ changes from the activated pinned vortex state to the vortex liquid state as the magnetic field increases, leading to two pairs of antisymmetric peaks in both *R*^*2ω*^*-B* and *V*_*DC*_*-B* curves at *T* ≥ 2 K (see Supplementary Note 2). It should also be noted that compared to conventional ratchets composed of artificial structures which rectify AC-driven vortices into a DC electric field without sensing ability^17,42,43^, our device utilizes the intrinsic inversion symmetry breaking in few-layer NbSe_2_ and provides extreme sensitivity in the superconducting regime, thus realizing nonreciprocal sensing in 2D NbSe_2_.

### Manipulating nonreciprocal sensitivity in NbSe_2_ antenna

**Fig. 5:**
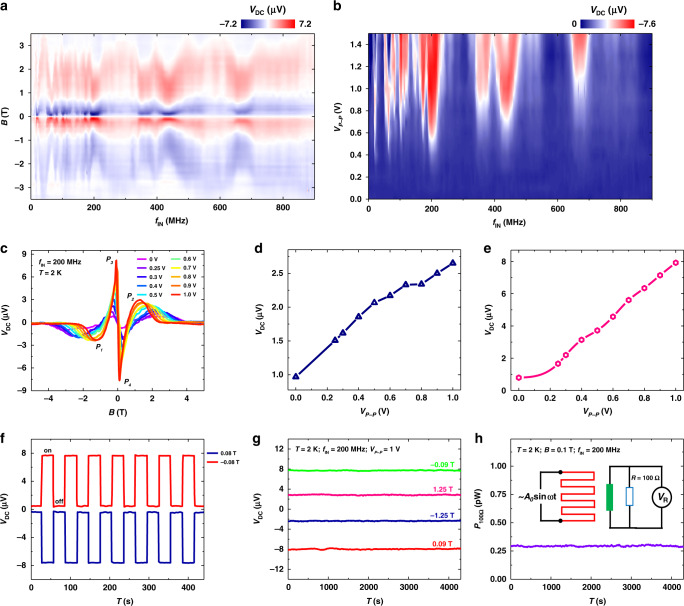
Manipulating nonreciprocal sensitivity in NbSe_2_ antenna. **a**, Color plot of *V*_*DC*_ as a function of the frequency *f*_*IN*_ and magnetic field of the NbSe_2_ antenna device at *V*_*P-P*_ = 1 V and *T* = 2 K. The NbSe_2_ thickness is five layers. **b**, Color plot of *V*_*DC*_ as a function of frequency *f*_*IN*_ and *V*_*P-P*_ of the device at *B* = 0.12 T and *T* = 2 K. **c**, *V*_*DC*_*-B* of the device with *V*_*P-P*_ value (that is, the power of the AC signal applied on the resistor) changing from 0 to 1 V at *T* = 2 K, *f*_*IN*_ = 200 MHz. *V*_*P*1_*-V*_*P*4_ are the peak values of *V*_*DC*_. **d**, **e**, Extracted *V*_*MAX1*_ and *V*_*MAX2*_ value as a function of *V*_*P-P*_ value, where $V_{MAX1} = \left( {\left| {V_{P1}} \right| + \left| {V_{P2}} \right|} \right)/2$, $V_{MAX2} = \left( {\left| {V_{P3}} \right| + \left| {V_{P4}} \right|} \right)/2$. Both *V*_*MAX1*_ and *V*_*MAX2*_ values increase monotonically as the increase of *V*_*P-P*_ value. **f**, The dynamic behavior of the device with the AC signal (*f*_*IN*_ = 200 MHz, *V*_*P-P*_ = 1 V) switching on and off at *B* = 0.08 T and −0.08 T, respectively. **g**, Retention behavior o*f* the device under various magnetic fields with a frequency of *f*_*IN*_ = 200 MHz, and *V*_*P-P*_ = 1 V at *T* = 2 K. **h**, Time evolution of the generated power of a 100 Ω resistor connected to the device (*B* = 0.1 T, *f*_*IN*_ = 200 MHz, *V*_*P-P*_ = 1 V, *T* = 2 K), showing a stable detection after 4300 s. Inset, a schematic circuit diagram of the measurement of the generated power on a 100 Ω resistor connected to the device.

To control the performance of the NbSe_2_ antenna device, we then try to change the frequency and the amplitude of the applied AC signal. Figure 5a displays the color plot of *V*_*DC*_ as a function of the frequency *f*_*IN*_ and magnetic field of the NbSe_2_ antenna device at *V*_*P-P*_ = 1 V and *T* = 2 K. The device shows prominent broadband sensing from 5 to 900 MHz(see Supplementary Figures 8, 9 for additional data) and the maximum response of the device appears at *f*_*IN*_ ∼ 200 MHz. Also, the color plot is symmetric with respect to y-axis and the sign of *V*_*DC*_ changes 3 times as the magnetic field increases from −3.5 to 3.5 T, indicating the nonreciprocal sensitivity of NbSe_2_ antenna device. Figure 5b shows the Color plot of *V*_*DC*_ as a function of frequency *f*_*IN*_ and *V*_*P-P*_ of the device at *B* = 0.12 T and *T* = 2 K. The entire spectrum agrees with Fig.5a and the device’s response increases with the increase of the *V*_*P-P*_, suggesting that the device provides an increased DC response as the applied power of the resistor increases (see Supplementary Fig. 8). This is also confirmed in Fig. 5c, which shows the DC response of the device under various AC amplitudes at *f*_*IN*_ = 200 MHz. Accordingly, as the amplitude increases from 0 to 1 V (considering the resistor value of ∼50 Ω, the applied power *P*_*IN*_ increases from 0 to 2.5 mW as *V*_*P-P*_ changes from 0 to 1 V, see Supplementary Fig. 8), the maximum value *V*_*MAX1*_ increases from 3.5 to 5.9 μV and *V*_*MAX2*_ increases from 0.5 to 22 μV (Fig. 5d, e). Here *V*_*MAX1*_ and *V*_*MAX2*_ are defined as $V_{MAX1} = \left( {\left| {V_{P1}} \right| + \left| {V_{P2}} \right|} \right)/2$ and $V_{MAX2} = \left( {\left| {V_{P3}} \right| + \left| {V_{P4}} \right|} \right)/2$, where *V*_*P*1_*-V*_*P*4_ are the peak values of *V*_*DC*_ as shown in Fig. 5a. The larger variation of *V*_*MAX2*_ than *V*_*MAX1*_ in response to increased applied power is due to the larger *γ´* value than *γ* (see Supplementary Note 2). All these results suggest that we have successfully pushed the relative low-frequency rectification in nonreciprocal charge transport into the radio-frequency sensitivity of the antenna device. It should be noted that the maximum response frequency of each NbSe_2_ device varies. This may due to the different levels of disorder and defects in each device (see Supplementary Figs. 11–15 for additional antenna devices with different thicknesses). Additionally, the device does not show a cutoff behavior at the maximum frequency that our equipment can reach (900 MHz). Note this value is much larger than the resonant frequency of the vortex moving in the artificial periodic potential (∼100 MHz)^18^. This may due to the much smaller size (atomic level) of the asymmetric pinning potential in 2D NbSe_2_(see Supplementary Note 4). Thus, the NbSe_2_ sensing antenna has great potential to realize sensitive detection at much higher frequencies in the future.

To probe the stability of the device, we first measure the dynamic behavior of the NbSe_2_ antenna device. In Fig. 5f, the DC response of the device was monitored with the AC signal (*f*_*IN*_ = 200 MHz, *V*_*P-P*_ = 1 V) being switching on and off. The device exhibits a stable and repeatable response to the AC signal with different magnetic fields and the on/off voltage ratio reaches ∼17. We then turn to measure the retention characteristic of the NbSe_2_ antenna. As shown in Fig. 5g, we keep the magnetic field at the peak position value (*P*_*1*_-*P*_*4*_ inset Fig. 5g) and measure the DC response of the device with the AC wave (*F*_*IN*_ = 200 MHz, *V*_*P-P*_ = 1 V) applied on the resistor. The DC response of the device was monitored for 4300 s, during which the generated DC voltage is very stable in all four states. This nonreciprocal multi-states of the antenna device suggest its potential applications in radio-frequency information storage and identification^44^. As shown in Fig. 5h, we have also connected a 100 Ω load resistor to the device (Fig. 5h inset) and measured the power on it (*P*_*L*_) for 4300 s. The generated power *P*_*L*_ by the NbSe_2_ antenna is very stable during the measurement. Note *P*_*L*_ here is a net value because we have subtracted the power generated from the environmental fluctuation. All these experiments suggest good device stability and versatile application capability of the NbSe_2_ sensing device in electronic circuits.

## Discussion

During the experiment, we have also performed second harmonic and DC response measurement on samples with various thicknesses (see Supplementary Figs. 11–15 for details). Due to the weak interlayer coupling, few-layer NbSe_2_ behaves like its monolayer rather than bulk material in which inversion symmetry is preserved. As a result, we can observe the nonreciprocal charge transport and DC sensitivity in few-layer NbSe_2_ which is not present in the bulk material. We believe that the nonreciprocity still dominates the vortex motion due to the weaker interlayer coupling constant compared to the spin splitting energy in few-layer NbSe_2_ (see Supplementary Note 7 for a detailed discussion). However, the underlying exquisite physics needs further theoretical and experimental investigation. This study has suggested that low-dimensional crystalline superconductors are promising systems to realize the control of vortex motion and further device applications. Our work highlights NbSe_2_ as a model system for exploring nonreciprocal charge transport and controlling nonreciprocal sensitivity in its antenna devices, paving the way to understanding the exotic physics in layered low-dimensional crystalline superconductors and allowing their integration into new functionality devices.

## Methods

Sample growth. High-quality 2H-NbSe_2_ single crystals were synthesized using the Chemical Vapor Transport (CVT) method. The stoichiometric-ratio of Nb and Se powders with 0.2% excess of Se and 0.1 g iodine were evacuated and sealed in the quartz tube. The sealed tube was then placed in a double zone furnace horizontally and grown for 2 weeks in a temperature gradient of 730 to 770 °C. After that, the single crystals of 2H-NbSe_2_ were formed at the low-temperature end.

Device fabrication. Different thicknesses of NbSe_2_ were obtained through mechanical exfoliation of bulk single crystals onto pre-patterned SiO_2_(285 nm)/Si substrates using polydimethylsiloxane (PDMS) stamps in the glove box. Multi-terminal electrical contacts were fabricated by standard EBL process using Polymethylmethacrylate/Methyl methacrylate bilayer polymer and subsequent deposition of Ti/Au (5 nm/80 nm) by magnetron sputtering. For the antenna device with the resistor, the resistance of the resistor was intentionally set to be ∼50 Ω by controlling the thickness of the gold and the length and width of the resistor. We limited the time that the devices were exposed to the air to less than 3 min to minimize the environmental effects on NbSe_2_.

Transport measurements. Four-terminal temperature-dependent magnetotransport and two-terminal DC measurements were carried out in a Physical Property Measurement System (PPMS) system (Quantum Design). Both the first- and second-harmonic signals of the AC resistance were measured by means of lock-in amplifiers (SR830) by applying an AC current $I = \sqrt 2 I_0\mathrm{sin}t ωt$. The measured first and second harmonic resistance is defined as *R*^*ω*^ *= V*^*ω*^*/I*_*0*_, *R*^*2ω*^ *= V*^*2ω*^*/I*_*0*_, here *I*_*0*_ is the effective value of the applied AC current, *V*^*ω*^ and *V*^*2ω*^ are the measured first harmonic and second harmonic voltage drop. During the AC resistance measurements, the applied current frequency is between 10–100 Hz. The phase between the first and second harmonic signal was set to be π/2. The DC response of the NbSe_2_ sensing device was collected using Keithley 2182 A and the high-frequency sine wave AC signal was applied to the resistor using Zurich Instruments UHFLI and Keithley 3390.

## Supplementary information

Supplementary Information

## Data Availability

The data that support the plots within this paper and other findings of this study are available from the corresponding author upon reasonable request.
