# Supplementary material for: Nonreciprocal superconducting NbSe2 antenna
Source: Nat Commun. 2020 Nov 6;11:5634. doi: 10.1038/s41467-020-19459-5 (PMC7648749; doi:10.1038/s41467-020-19459-5)
Supplement: Supplementary file 1 — Supplementary Information [file 41467_2020_19459_MOESM1_ESM.pdf]

Supplementary Information for

Nonreciprocal Superconducting $NbSe_2$ Antenna

Enze Zhang[1,2], Xian Xu[3], Yi-Chao Zou[4], Linfeng Ai[1,2], Xiang Dong[1], Ce Huang[1,2], Pengliang Leng[1,2], Shanshan Liu[1,2], Yuda Zhang[1,2], Zehao Jia[1,2], Xinyue Peng[1,2], Minhao Zhao[1,2], Yunkun Yang[1,2], Zihan Li[1,2], Hangwen Guo[2], Sarah J. Haigh[4], Naoto Nagaosa[5,6], Jian Shen[1,2,7,8], Faxian Xiu[1,2,7,8]*

[1]State Key Laboratory of Surface Physics and Department of Physics, Fudan University, Shanghai 200433, China

[2]Institute for Nanoelectronic Devices and Quantum Computing, Fudan University, Shanghai 200433, China

[3]School of Physics Science and Engineering, Tongji University, Shanghai 200092, China

[4]School of Materials, University of Manchester, Manchester M13 9PL, United Kingdom

[5]Department of Applied Physics, The University of Tokyo, Tokyo 113-8656, Japan

[6]RIKEN Center for Emergent Matter Sciences (CEMS), Saitama 351-0198, Japan

[7]Collaborative Innovation Center of Advanced Microstructures, Nanjing University, Nanjing 210093, China

[8] Shanghai Research Center for Quantum Sciences, Shanghai 201315, China

E-mail: Faxian@fudan.edu.cn

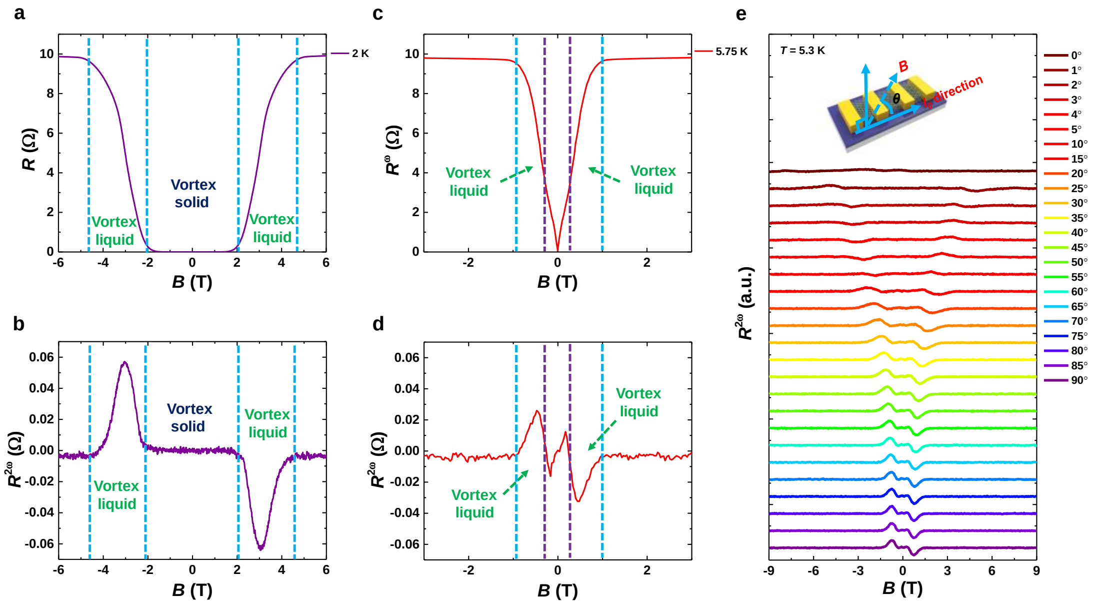

Supplementary Figure 1| Additional data from the five-layer $NbSe_2$ device shown in the main text. a,b, R^{ω}-B and $R^{2\omega}$-B curves of the five-layer $NbSe_2$ device from the main text at $T = 2$ K where different states are marked. The vortex solid (glass) state corresponds to zero R^{ω} and zero $R^{2\omega}$ regimes. The vortex liquid state corresponds to nonzero R^{ω} and nonzero $R^{2\omega}$ regime. **c,d,** R^{ω}-B and $R^{2\omega}$-B curves of the device at $T =$ 5.75 K. The purple dashed lines define the boundary between the activated vortex state at relatively high temperature and the vortex liquid state defined as the magnetic field ($B \neq 0$) where the $R^{2\omega}$ sign is reversed. In other words, the activated vortex state is the regime between the two purple dash lines in **c** and **d**. **e.** Angular dependent $R^{2\omega}$-B curves of the five-layer device as the magnetic field changes (as schematically shown in inset) from the out-of-plane direction (perpendicular to the substrate direction) to the in-plane direction (parallel to the current direction). The peak values of the $R^{2\omega}$-B curves gradually disappear when the magnetic field is parallel to the current direction.

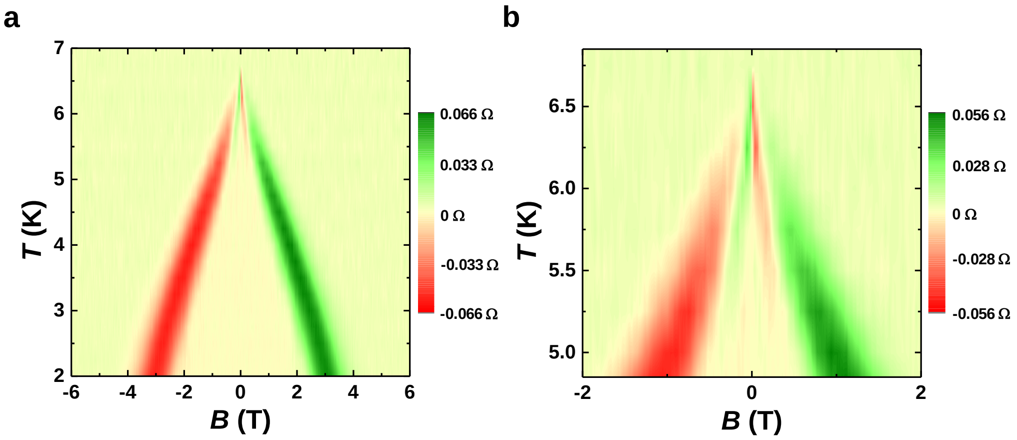

Supplementary Figure 2| 2D plot of the second harmonic resistance of the five-layer $NbSe_2$ device in the main text. a,b, Large and small range 2D plots of the second harmonic resistance of the five-layer $NbSe_2$ device in the main text. The second harmonic signal has a different sign under positive and negative magnetic fields at $T \leq 5$ K(a), another pair of peaks emerge at $T \geq 5.25$ K (b).

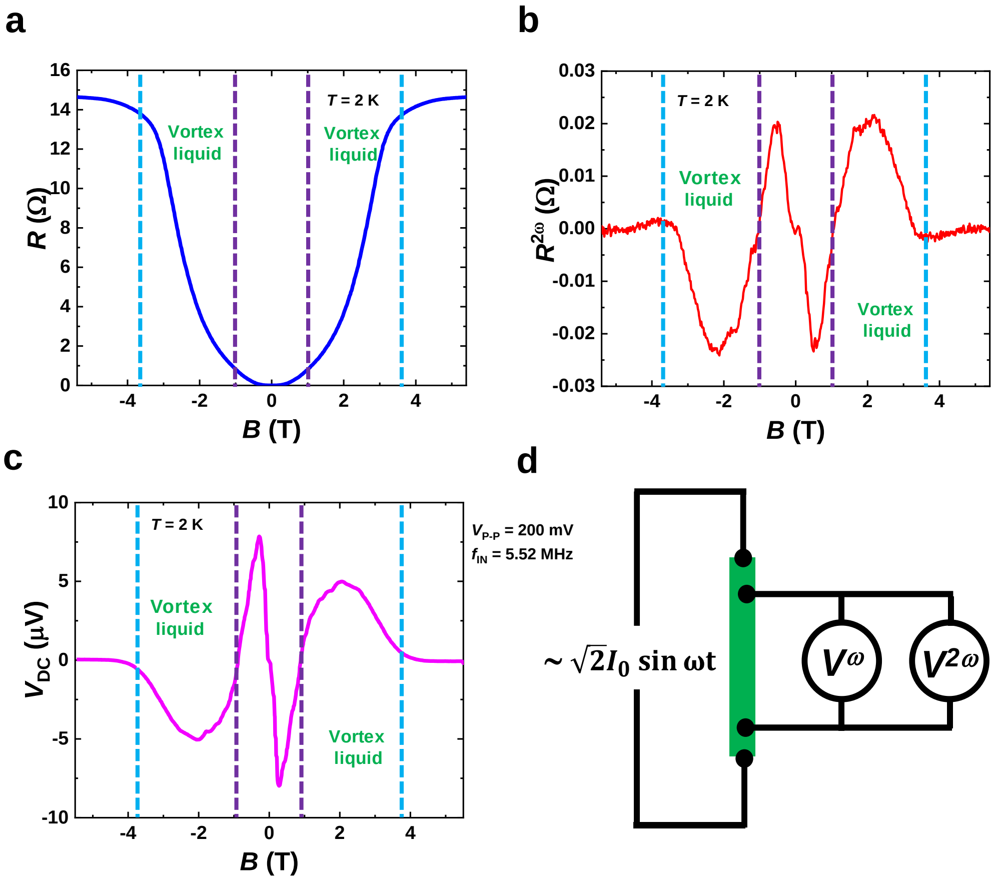

Supplementary Figure 3| Nonreciprocal charge transport and the *d.c.* response of the trilayer $NbSe_2$ device in the main text. a, b, c, R^{ω}-B, $R^{2\omega}$-B and V_{DC}-B of the trilayer device at T = 2 K. The $R^{2\omega}$-B and V_{DC}-B curves exhibit two pairs of peaks, indicating no vortex solid states at T = 2 K. This is also consistent with the nonzero magnetoresistance in the R^{ω}-B curve at $B \neq 0$ T in **a**. Accordingly, the activated vortex state and vortex liquid state are separated by the two dashed purple lines corresponding to the magnetic field where R^{ω} changes its sign. The absence of the vortex solid state in the trilayer $NbSe_2$ device at T = 2 K is because the melting temperature of thin $NbSe_2$ is lower than that in thicker samples[1]. **d,** Schematic circuit diagram of the measurement of the R^{ω} and $R^{2\omega}$. Both the first- and second-harmonic signals of the *a.c.* resistance were measured using lock-in amplifiers by applying an *a.c.* current $I = \sqrt{2}I_0 sin\omega t$. The measured first and second harmonic resistance is defined as $R^{\omega} = V^{\omega}/I_0$, $R^{2\omega} = V^{2\omega}/I_0$. Here I_0 is the effective value of the applied *a.c.* current, V^{ω} and $V^{2\omega}$ are the measured first harmonic and second harmonic voltage drop.

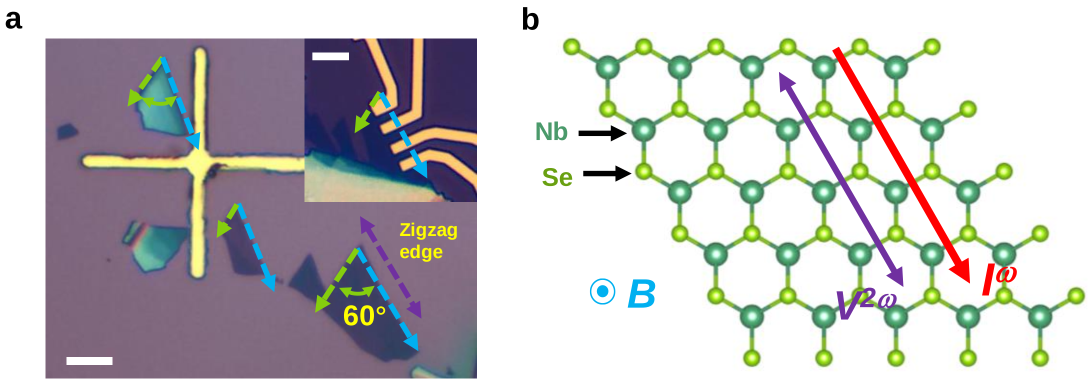

Supplementary Figure 4| Details about the direction of the electrical contacts and measurement configuration. a, Optical image of the cleavage edges of the exfoliated $NbSe_2$ crystals. The cleavage edges have a high probability to form a 60° angle as marked by the dashed green and light blue arrows. The purple arrow stands for the zigzag edge of the $NbSe_2$ crystal. Scale bar, 10 μm. Inset, an optical image of the fabricated device. The electrical contacts are along the cleavage edge (zigzag edge). Scale bar, 5 μm. **b,** Schematic image of the measurement configuration. The magnetic field is in the out-of-plane direction. The applied current is in the zigzag direction. The longitudinal nonreciprocal response ($V^{2\omega}$, the purple arrow) is maximum under such a measurement configuration.

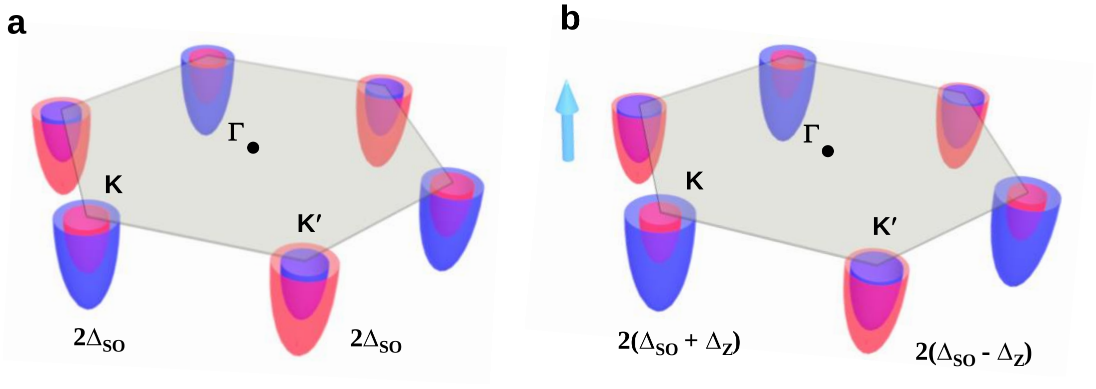

Supplementary Figure 5| Schematic Fermi surface of $NbSe_2$ with and without perpendicular magnetic field. a, b, Schematic Fermi surface of $NbSe_2$ with (left, **a**) and without (right, **b**) perpendicular magnetic field. Δ_{SO} is the spin splitting, Δ_Z is the Zeeman splitting. The band asymmetry appears between the K and K′ points due to the synergetic effect of the spin-orbit interaction and Zeeman effect. Their spin splittings are $2(\Delta_{SO} + \Delta_Z)$ and $2(\Delta_{SO} - \Delta_Z)$, respectively[2,3]. At zero magnetic field, the lack of inversion symmetry manifests itself as the spin splitting of bands due to the spin-orbit interaction. Applying the magnetic field breaks the time-reversal symmetry, the band asymmetry appears between the K and K′ points due to the synergetic effect of the spin-orbit interaction and Zeeman effect. The spin splittings are $2(\Delta_{SO} + \Delta_Z)$ and $2(\Delta_{SO} - \Delta_Z)$ at K and K′ points, respectively. Therefore, the nonreciprocal current responses in 2D $NbSe_2$ arise when both inversion and time-reversal symmetries are broken.

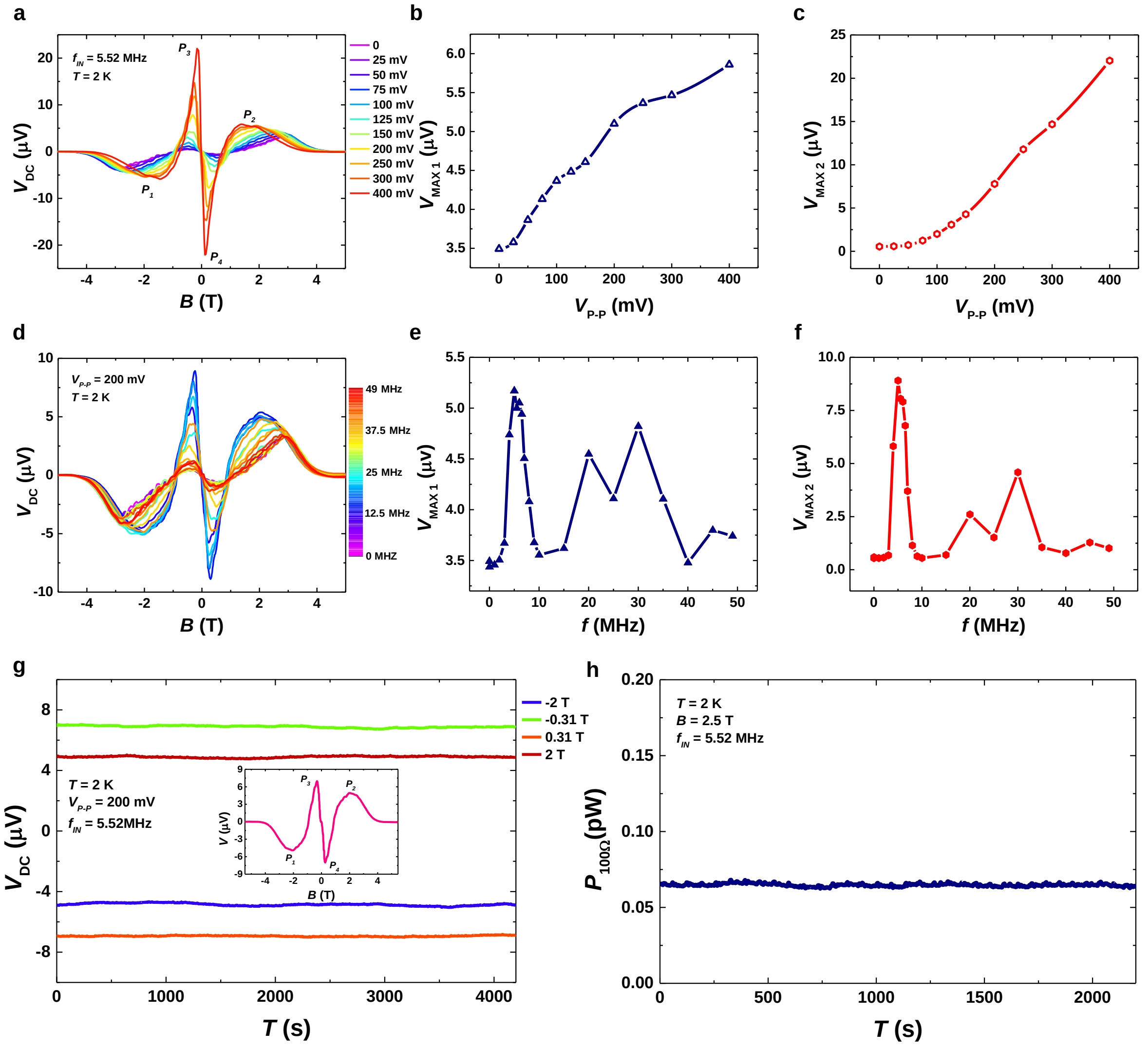

Supplementary Figure 6| Nonreciprocal sensitivity of the thrlayer $NbSe_2$ antenna device in the main text. a, V_{DC}-B of the device under *a.c.* signal applied on the resistor with V_{P-P} value changing from 0 to 400 mV at T = 2 K. V_{P1}-V_{P4} are the peak values. **b,c,** Extracted V_{MAX1} and V_{MAX2} value as a function of V_{P-P} value. Both V_{MAX1} and V_{MAX2} values increase monotonically as the increase of V_{P-P} value (that is, the applied power on the resistor). **d,** V_{DC}-B of the device under *a.c.* signal with the frequency changing from 0 to 49 MHz. V_{DC} shows a distinguishable increase with $f_{IN} \geq 1$ MHz. **e,f,** Extracted V_{MAX1} and V_{MAX2} values as a function of frequency f_{IN}, showing the largest response at f_{IN} ~5.52 MHz. **g,** Retention behavior of the device under various magnetic fields with a frequency of f_{IN}= 5.52 MHz, and V_{P-P} = 200 mV at T = 2 K. Inset, V_{DC}-B of the devices with f_{IN} = 5.52 MHz and V_{P-P} = 200 mV at T = 2 K, where P_1-P_4 positions are marked. **h,** Time evolution of the generated power of a 100 Ω resistor connected to the device (B = 2.5T, f_{IN} = 5.52 MHz, V_{P-P} = 200 mV, T = 2 K), showing a stable detection after 2200 seconds.

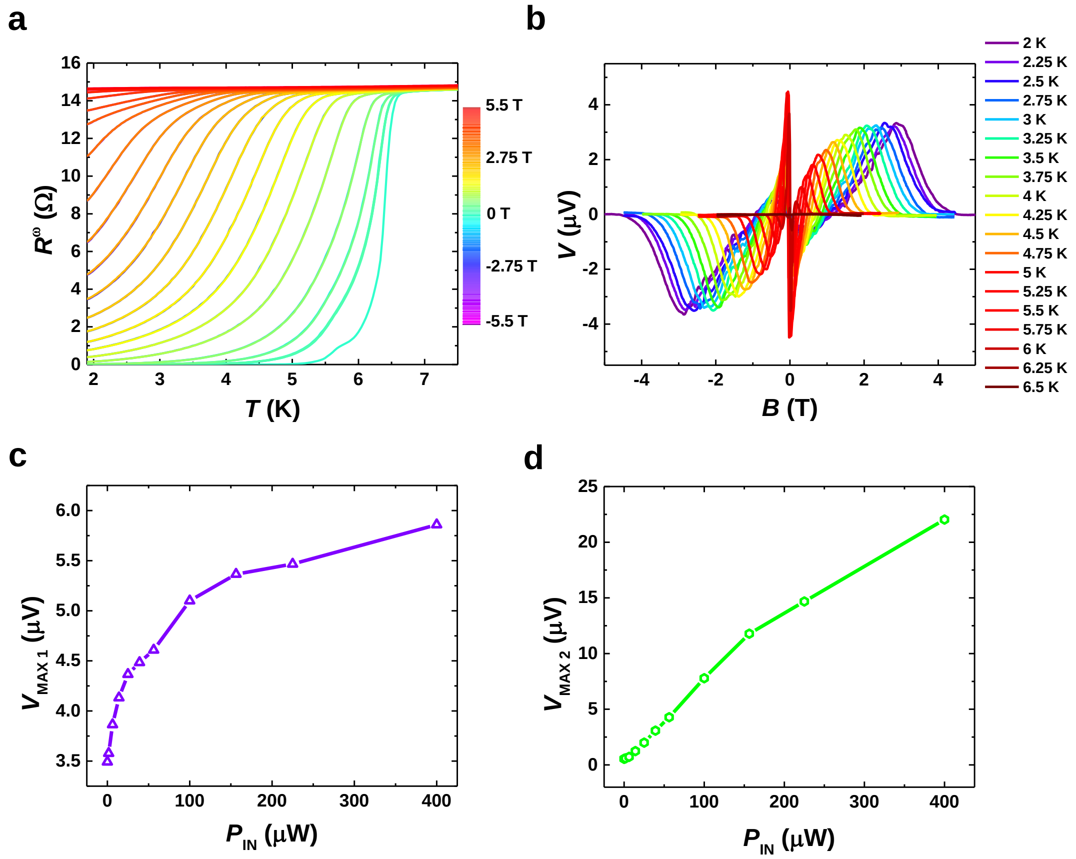

Supplementary Figure 7| Additional data of the trilayer $NbSe_2$ device in the main text. a, Temperature-dependent first harmonic resistance under applied magnetic field varying from -5.5 to 5.5 T, where each pair of $R^{\omega}(B)$-T and $R^{\omega}(-B)$-T curves overlap each other. **b,** The *d.c.* response of the trilayer device as a function of *B* with no *a.c.* signal applied on the resistor in the main text Fig. 4b as the temperature changes from 2 to 6.5 K. The curve also shows a similar behavior as the main text Fig. 4f due to the extreme sensitivity of the $NbSe_2$ device to the environmental fluctuation (The PPMS chamber has a noise background of a few MHz[4]) in the superconducting regime. **c,d,** V_{MAX1} and V_{MAX2} as a function of the input power applied on the resistor (The data are obtained from the Supplementary Figure 6a). The device shows an increased *d.c.* response at the input power increased from 0 to 400 μW. Also, the device shows a response at P_{IN} = 1.56 μW, indicting its high sensitivity to the applied *a.c.* electromagnetic wave.

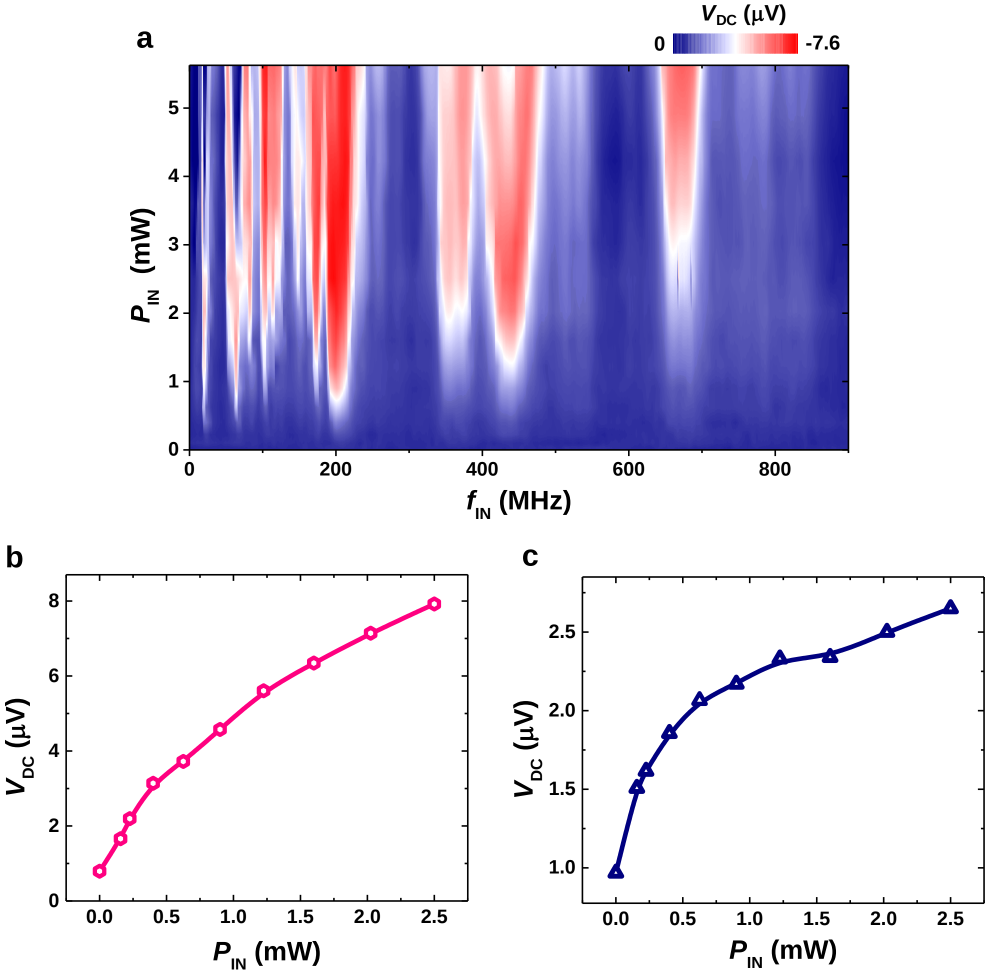

Supplementary Figure 8| Power-dependent d.c. response of the five-layer $NbSe_2$ antenna device in the main text. a, Color plot of V_{DC} as a function of frequency f_{IN} and P_{IN} of the device at B = 0.12 T and T = 2 K, the resistor was placed 2 mm away from the $NbSe_2$ channel. **b,c,** V_{MAX1} and V_{MAX2} as a function of the input power applied on the resistor (The data are obtained from the main text Figure 5a). Both V_{MAX1} and V_{MAX2} increases as the input power increased from 0 to 2.5 mW.

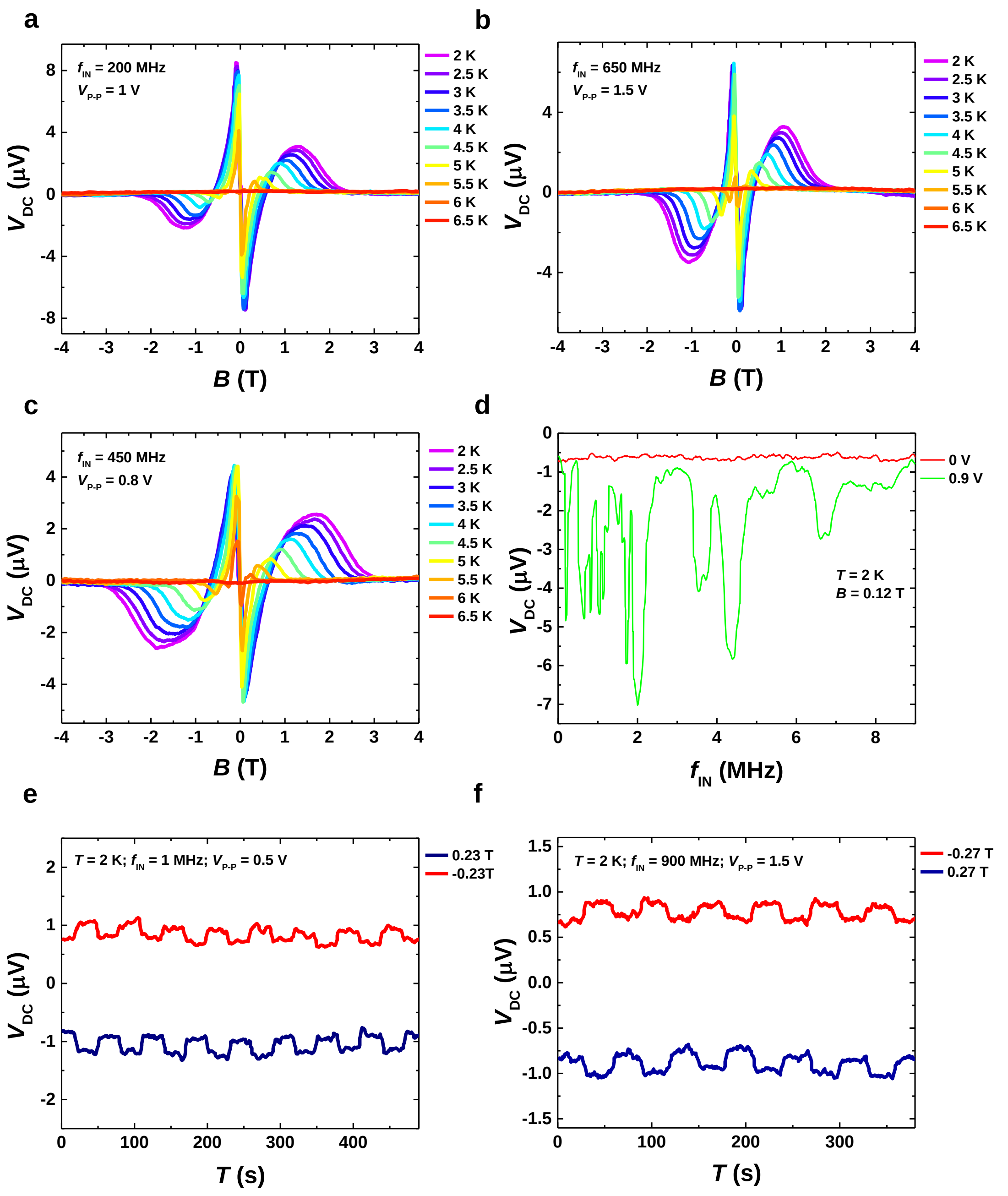

Supplementary Figure 9| Additional data of the five-layer $NbSe_2$ antenna device in the main text. a-c, The *d.c.* response of the device at 2 K ≤ T ≤ 6.5 K with applied *a.c.* signals (**a**, f_{IN} = 200 MHz, $V_{P\text{-}P}$ = 1.5 V; **b**, f_{IN} = 450 MHz, $V_{P\text{-}P}$ = 1.5 V; **c**, f_{IN} = 650 MHz, $V_{P\text{-}P}$ = 1.5 V;) on the resistor, all of them shows prominent d.c. response. Also, Increasing the temperature would weaken the d.c. response. **d**, V_{DC} as a function of f_{IN} at $V_{P\text{-}P}$ = 0 V and 0.9 V, respectively. The device shows prominent d.c. response at f_{IN} ≥ 5 MHz. The maximum response occurs at f_{IN} = 200 MHz. **e,** Dynamic behavior of the device with the *a.c.* signal (f_{IN} = 1 MHz, $V_{P\text{-}P}$ = 0.5 V) switching on and off at B = 0.23 T and -0.23 T, respectively. **f,** Dynamic behavior of the device with the *a.c.* signal (f_{IN} = 900 MHz, $V_{P\text{-}P}$ = 1.5 V) switching on and off at B = 0.27 T and -0.27 T, respectively.

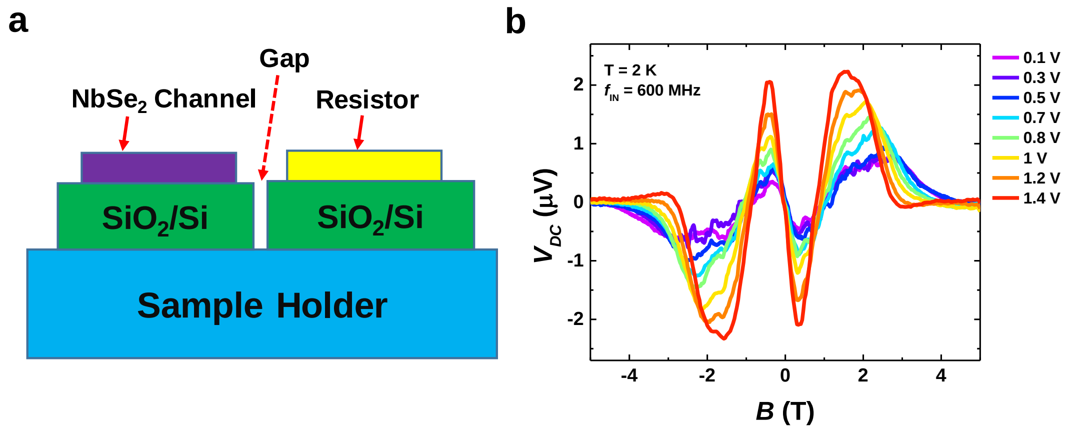

Supplementary Figure 10| a, Schematic figures of the control experiment, the $NbSe_2$ channel and resistors are fabricated on different SiO2/Si channels. There is a gap (marked by the red dashed arrow) between these two wafers so as to block out the possible heating effect. **b,** V_{DC}-B of the device under various $V_{P\text{-}P}$ value (that is, the power of the a.c. signal applied on the resistor) at T = 2 K, f_{IN} = 600 MHz.

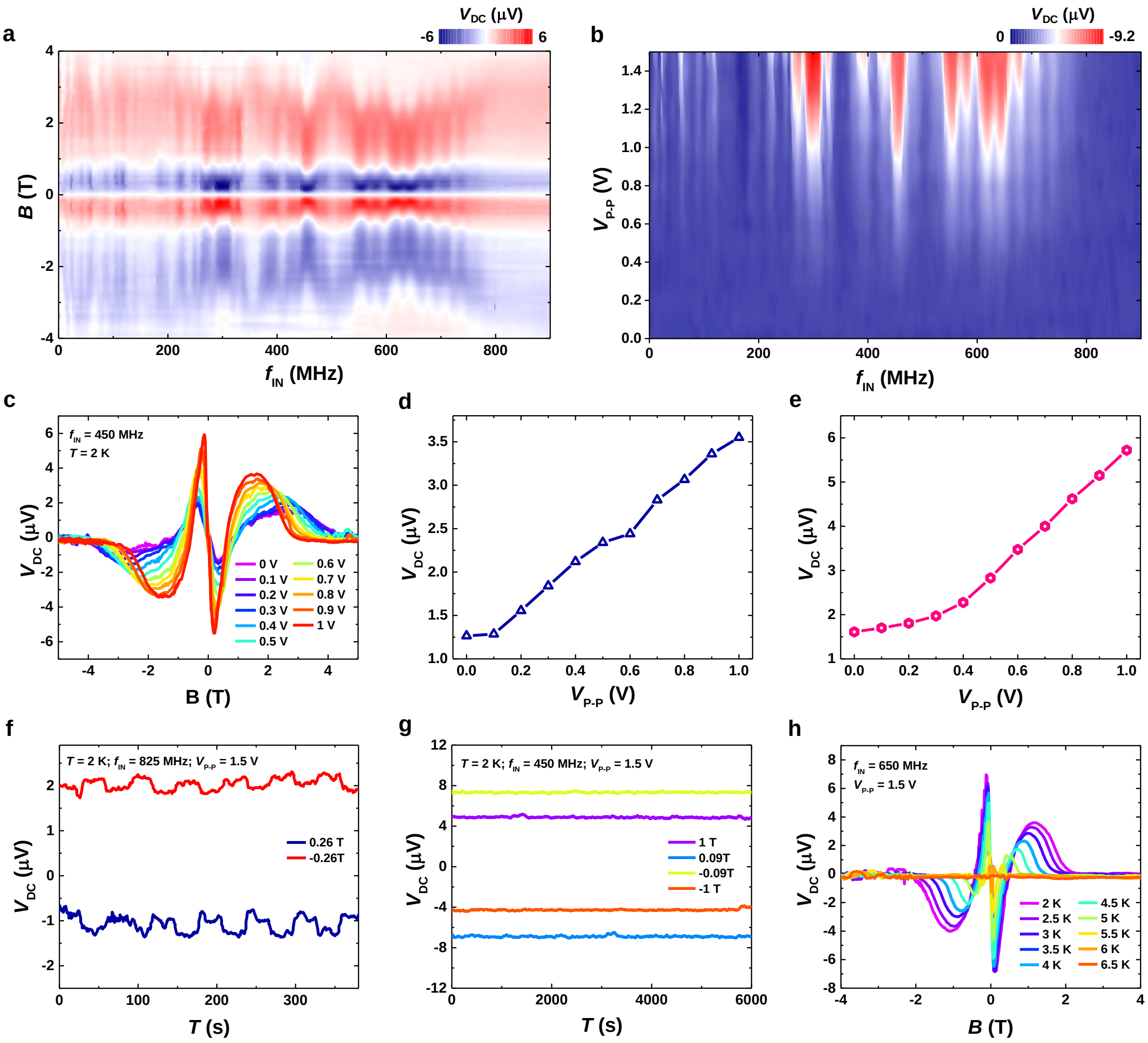

Supplementary Figure 11| Additional data for another six-layer $NbSe_2$ antenna device. a, Color plot of V_{DC} as a function of the frequency f_{IN} and magnetic field of the $NbSe_2$ antenna device at $V_{P\text{-}P}$ = 1 V and T = 2 K. The $NbSe_2$ thickness is six layers. **b,** Color plot of V_{DC} as a function of frequency f_{IN} and $V_{P\text{-}P}$ of the device at B = 0.12 T and T = 2 K. **c,** V_{DC}-B of the device with $V_{P\text{-}P}$ value (that is, the power of the a.c. signal applied on the resistor) changing from 0 to 1 V at T = 2 K, f_{IN} = 450 MHz. **d, e,** Extracted V_{MAX1} and V_{MAX2} value as a function of $V_{P\text{-}P}$ value, Both V_{MAX1} and V_{MAX2} values increase monotonically as the increase of $V_{P\text{-}P}$ value. **f,** The dynamic behavior of the device with the *a.c.* signal (f_{IN} = 825 MHz, $V_{P\text{-}P}$ = 1V) switching on and off at B = 0.08 T and -0.08 T, respectively. Considering the color plot in **a** and **b**, the device shows a d.c. response from 1 to 825 MHz. **g,** Retention behavior of the device under various magnetic fields with a frequency of f_{IN} = 450 MHz, and $V_{P\text{-}P}$ = 1V at T = 2 K. **h,** $R^{2\omega}$-B curves of the device with the temperature changing from 2 to 6.75 K at f_{IN} = 650 MHz, $V_{P\text{-}P}$ = 1.5V.

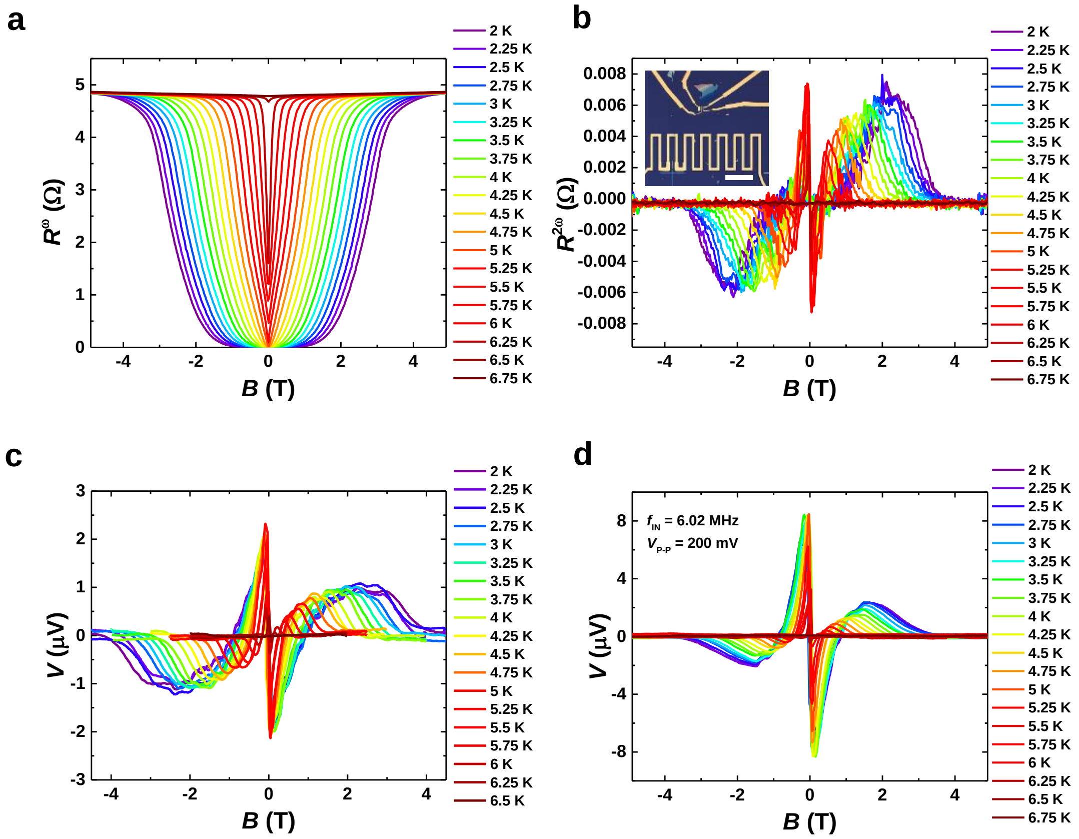

Supplementary Figure 12| Additional data for a four-layer $NbSe_2$ antenna device. **a,** Magnetoresistance isotherms of the device at temperatures ranging from 2 to 6.5 K. **b,** Corresponding $R^{2\omega}$-B curve of the device as the temperature varies from 2 to 6.5 K, showing an antisymmetric behavior (the applied current is $I_0 = 50$ μA). Inset, an optical image of the device. Scale bar, 50 μm. **c,** the *d.c.* response of the device at 2 K ≤ T ≤ 6.5 K without applying *a.c.* signals on the resistor, showing a similar antisymmetric behavior to the $R^{2\omega}$-B curve. **d,** The *d.c.* response of the device at 2 K ≤ T ≤ 6.5 K under applied *a.c.* signal on the resistor (F_{IN} = 6.02 MHz, $V_{P\text{-}P}$ = 200 mV), consistent with the trilayer device in the main text.

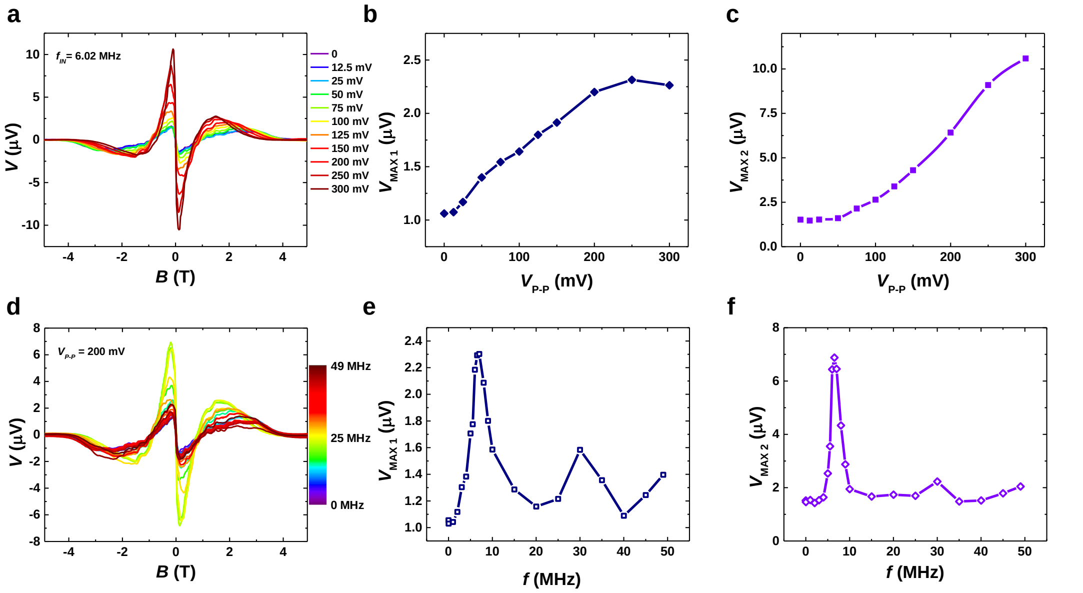

Supplementary Figure 13| Controlling of the *d.c* response of the four-layer $NbSe_2$ antenna device. a, V_{DC}-B of the device with V_{P-P} values of the *a.c.* signal applied on the resistor changing from 0 to 200 mV at T = 2 K. **b,c,** Extracted V_{MAX1} and V_{MAX2} values as a function of V_{P-P} value**,** V_{MAX1} and V_{MAX2} are the same as defined in the main text. V_{P1}-V_{P4} are the peak values of V_{DC} as shown in a. **d,** V_{DC}-B of the device under *a.c.* signal frequency changing from 0 to 49 MHz, showing an increased V_{DC} as $f_{IN} \geq 1$ MHz**. e,f,** Extracted V_{MAX1} and V_{MAX2} values as a function of *a.c.* signal frequency f_{IN} value, showing the largest response at f_{IN} ~ 6.02 MHz.

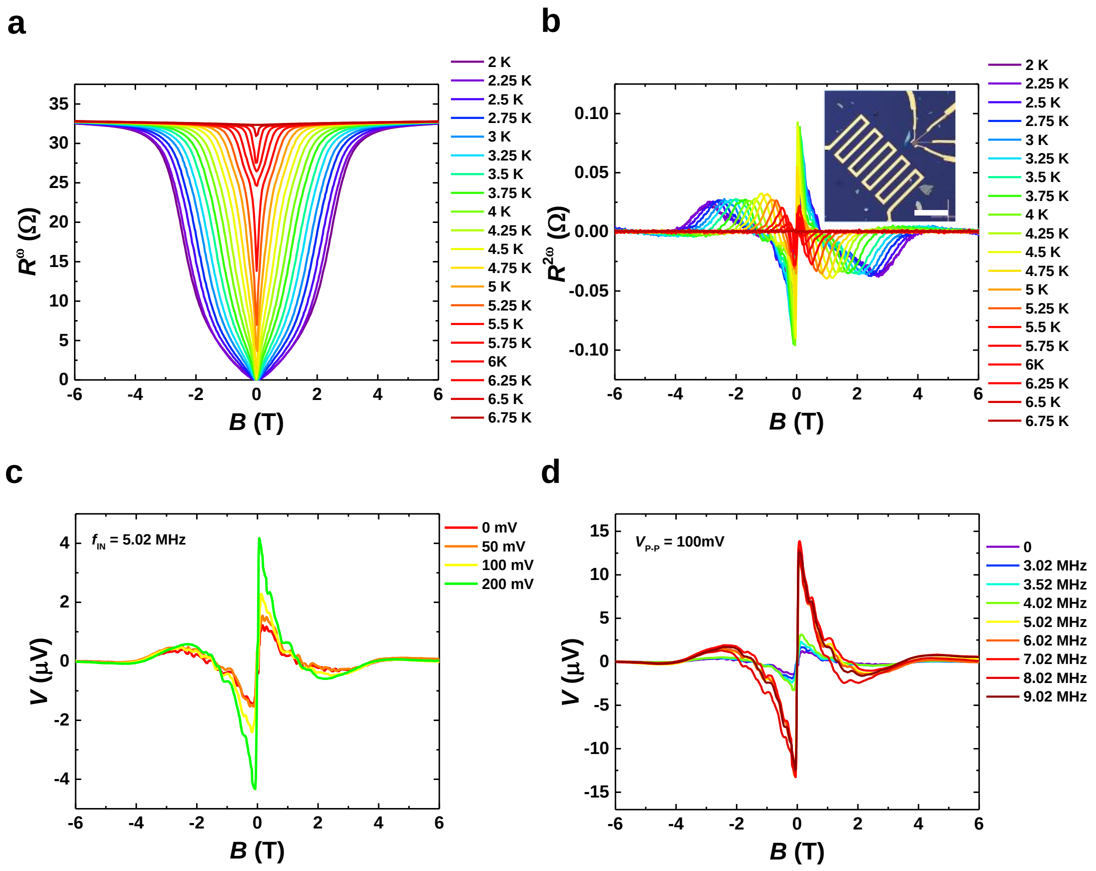

Supplementary Figure 14| Second harmonic and *d.c.* response measurement of double-layer $NbSe_2$. a, Magnetoresistance isotherms of the double-layer device at various temperatures. **b,** Corresponding $R^{2\omega}$-B curve of the double-layer device with applied current I_0 = 15 μA, exhibiting two pairs of antisymmetric peaks. Inset, an optical image of the device. Scale bar, 50μm. **c,** V_{DC}-B curves of the device at T = 2 K under *a.c.* current applied to the resistor. The current amplitude $V_{P\text{-}P}$ varies from 0 to 200 mV (f_{IN} = 5.02 MHz). V_{DC} increases as the increase of applied current amplitude $V_{P\text{-}P}$. **d,** V_{DC}-B curve of the double-layer device under *a.c.* current ($V_{P\text{-}P}$ = 100 mV) applied on the resistor with the frequency varying from 0 to 9.02 MHz, showing a maximum response at f_{IN} = 5.02 MHz.

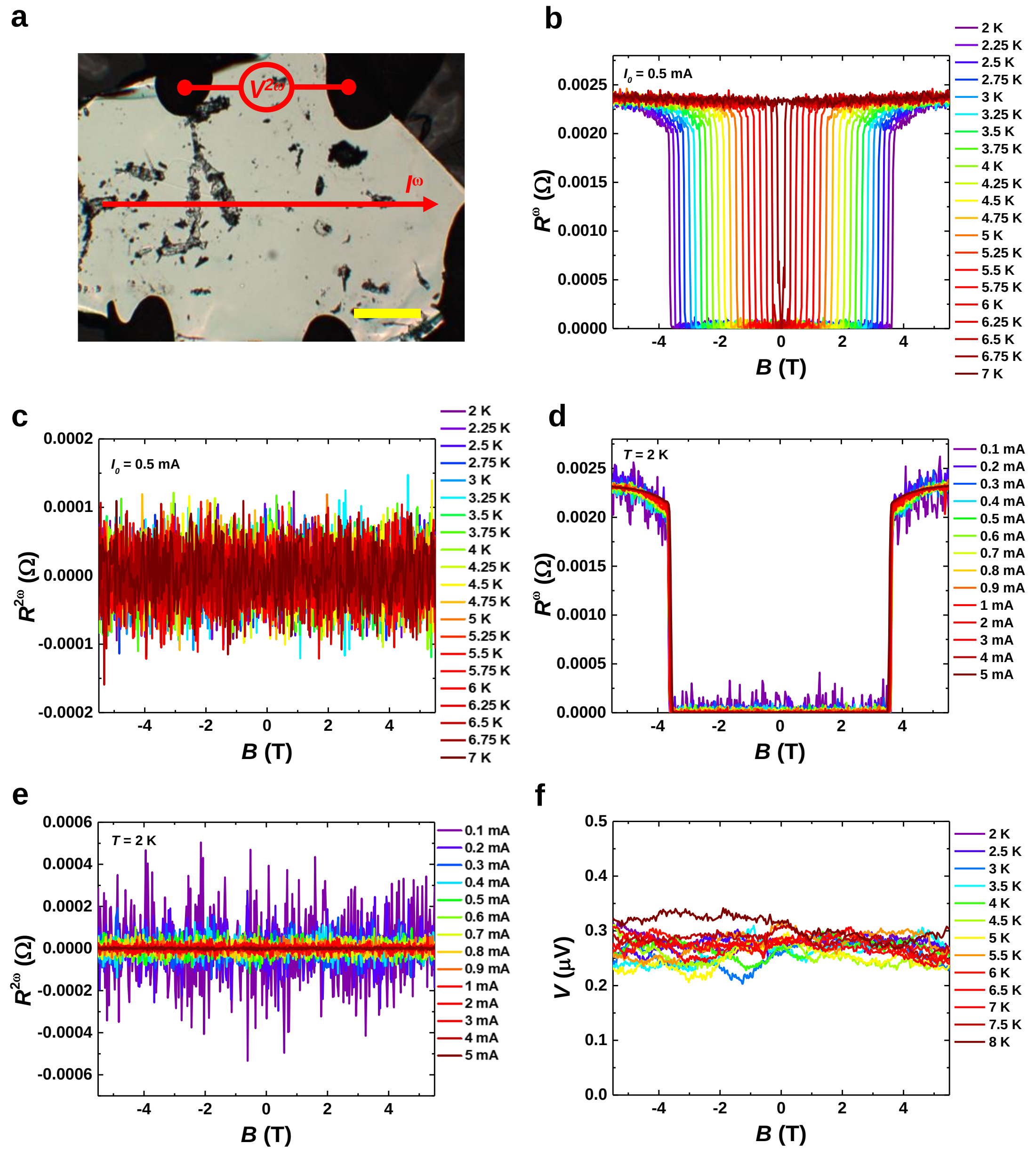

Supplementary Figure 15| Second harmonic and *d.c.* response measurement of bulk $NbSe_2$. a, Optical image of the bulk $NbSe_2$ four-terminal device, where the applied current direction has been marked (red solid curve). Scale bar, 1mm. **b,** Magnetoresistance isotherms of the bulk device with temperatures from 2 to 8 K. The applied current is 0.5 mA. **c,** Corresponding $R^{2\omega}$-B curve of the bulk device, showing no signal at all temperatures from 2 to 8 K. **d,** R^{ω}-B curves of the bulk device at $T = 2$ K under applied currents from 0.1 to 5 mA. **e,** Corresponding $R^{2\omega}$-B curve of the bulk device under various applied currents, also showing no signal. **f,** The *d.c.* response of the bulk device without applying the *a.c.* signal, signaling no response to the environment fluctuation.

Supplementary Note 1. A detailed derivation of equations (1) and (2) in the main text.

Considering the second-order Taylor expansion formula in two variables:

$$f(x_0+h,y_0+k) = f(x_0,y_0)+\left(h\frac{\partial}{\partial x}+k\frac{\partial}{\partial y}\right)f(x_0,y_0)+\frac{1}{2!}\left(h\frac{\partial}{\partial x}+k\frac{\partial}{\partial y}\right)^2 f(x_0,y_0) + R_2$$

Here we set:

$$(x_0,y_0)\to(0,0) \qquad h\to I \qquad k\to B \qquad f\to R$$

Because *R(I, B)=R(-I, -B)*, then the first-order term is 0. Using a Taylor expansion at (0,0) and ignoring higher-order terms, we get:

$$R(I,B)=R(0,0)+\frac{1}{2!}\left(I\frac{\partial}{\partial I}+B\frac{\partial}{\partial B}\right)^2 R(I,B)$$
$$=R(0,0)+\frac{1}{2}\left[I^2\frac{\partial^2}{\partial I^2}R(I,B)+B^2\frac{\partial^2}{\partial B^2}R(I,B)+2IB\frac{\partial}{\partial I}\frac{\partial}{\partial B}R(I,B)\right].$$

Then we can set:

$$\alpha R_0=\frac{1}{2}\frac{\partial^2}{\partial I^2}R(I,B);$$

$$\beta R_0 B^2=\frac{1}{2}\frac{\partial^2}{\partial B^2}R(I,B);$$

$$\gamma R_0 B=\frac{\partial}{\partial I}\frac{\partial}{\partial B}R(I,B).$$

Here R_0 is the resistance at zero magnetic field, *I* represents the electrical current and *B* stands for the applied external magnetic field. Thus, we get the Taylor expansion of the resistance[5,6]:

$$R(I,B)=R_0(1+\alpha I^2+\beta B^2+\gamma IB) \qquad (1)$$

For systems exhibiting inversion symmetry[7],

$$R(I,B)=R(-I,-B).$$

Thus, the last term in equation (1) only exists in systems with broken inversion symmetry. Then the resistance of systems with inversion symmetry breaking can be described as:

$$R(I,B)=R_0(1+\gamma BI) \qquad (2)$$

If we apply a sine wave current $I=\sqrt{2}I_0\sin\omega t$ on the device, then the total resistance of the conductor is[7,8]:

$$V=\sqrt{2}R_0I_0\sin\omega t+\gamma BR_0I_0^2\sin\left(2\omega t-\frac{\pi}{2}\right)+\gamma BR_0I_0^2 \qquad (3)$$

Here the first term is the normal first harmonic signal, the second term is the second harmonic signal which has a π/2 phase shift comparing to the first harmonic signal, and the third term is the *d.c.* term that is usually neglected. Note that our research focuses on both the second term and the third term in equation (3). The *d.c.* term in

equation (3) can also be interpreted from the point of view of vortex flow in asymmetric pinning potentials, where the average *d.c.* voltage V_{DC} in response to an oscillatory *a.c.* drive is[9]

$$V_{DC} = \frac{1}{T}\int_0^T V(t)dt. \tag{4}$$

In the entire period T, $|V(t)|$ would have a larger value in the one-half period than the other half due to the asymmetric pinning potentials. Then V_{DC} will be nonzero.

At the same time, using the first term in equation (3), we get the first harmonic resistance,

$$R^{\omega} = R_0;$$

and the second-harmonic resistance

$$R^{2\omega} = \frac{\gamma B R_0 I_0}{\sqrt{2}}.$$

Then we can get the γ value as

$$\gamma = \frac{\sqrt{2}R^{2\omega}}{B R^{\omega} I_0},$$

which is the parameter representing the strength of the magneto-chiral anisotropy effect. Nevertheless, the $R^{2\omega}$ contains the B term, so if the magnetic field is reversed then the $R^{2\omega}$ will change sign. Therefore, the $R^{2\omega}$-B curve should be antisymmetric with respect to the *y*-axis, consistent with our experimental results.

Supplementary Note 2. The relation of nonreciprocal change transport and *d.c.* response with vortex motion.

For type-II superconductors like $NbSe_2$, the movement of the vortex causes dispassion in the superconducting states, leading to a nonzero resistance[10,11]. Compare Supplementary Fig. 1a with 1b, we find that the second harmonic signal is non-zero only at the intermediate states (the vortex flow regime in Supplementary Fig. 1a). The same phenomenon happens at all temperatures below T_C, suggesting a close relationship between the $R^{2\omega}$ and vortex motion. According to previous theories, in the vortex flow regime, the vortices driven by the external charge current moving among the asymmetric pinning potentials in non-centrosymmetric superconductors causes the emergence of nonreciprocal charge transport[2]. In 2D $NbSe_2$, the asymmetric pinning potentials naturally appear as a consequence of disorder[2] such as defects in 2D crystals[12,13] (Fig.1c in the main text) with inversion symmetry breaking. This is also the reason we using equation (1) to describe the nonreciprocal transport in 2D $NbSe_2$. During our second harmonic resistance measurement, when the temperature is low ($T \leq 5$ K), in the small magnetic field regime, the Lorentz force cannot overcome the pinning potential, therefore the vortex will not move[14]. In this case, there is no dissipation thus no magnetoresistance of $NbSe_2$. We call this state the vortex solid state or the vortex glass state[1]. While in a large magnetic field regime, Lorentz force starts to get larger than the pinning potential and the vortices can move freely in this regime. We call this state the vortex liquid state. Further increase of the magnetic field will

quench the superconductivity into the normal state, leading to a decrease of $R^{2\omega}$. When the temperature is further increased and finally reaches a critical point T_M (T_M = 5.25 K for the five-layer device in the main text), the pinned vortices will be thermally activated and jump between asymmetric pinning barriers. We call this state the activated pinned vortex state[15]. Note that the movement of the vortices in the activated pinned vortex state is called thermally-assisted flux flow[16], different from the vortex motion in the vortex liquid state. Then, both the activated pinned vortex state and vortex liquid state belong to the vortex flow regime. Accordingly, above the melting temperature, the system will change from the activated pinned vortex state to the vortex liquid state as the magnetic field increases. We believe that nonreciprocity of the vortex motion in the activated pinned vortex state and vortex liquid state are opposite, so the $R^{2\omega}$ will change its sign as we further increase the magnetic field regime at $T \geq T_M$. As a result, the $R^{2\omega}$-B curve exhibits two pairs of peaks at $T \geq 5.25$ K (as shown in Supplementary Figs. 1d, 2 and 3). Also, the T_M can reduce quite a lot as the thickness of the $NbSe_2$ reduces. This explains the phenomena where the $R^{2\omega}$-B curve exhibits two pairs of peaks at $T \geq 2$ K for the device in the main text Figure 4 (also see Supplementary Figs. 3b and c). Because the second harmonic signal and the *d.c.* term carry the same γB term (equation (2) above), the V_{DC}-B curve also shows two pairs of peaks quite similar to the $R^{2\omega}$-B curve (Supplementary Fig. 3c below). We note that the sign reversal in a vortex ratchet has been reported previously for Nb film grown on an array of Ni triangles[17] and superconducting Al film with an array of asymmetrical pinning sites[18]. The first case was interpreted as the effect of opposite ratchet motion of pinned vortex and interstitial vortex. The later one was explained by multiple reversals of vortex drift direction due to the increase of vortex density. Also, multi-reversal antisymmetric second harmonic magnetoresistance has been observed in a chiral WS_2 nanotube[7], in which the multi-reversal was due to the different chirality of the multiwall nanotube. The exquisite physics behind the two pairs of peaks observed in $R^{2\omega}$-B and V_{DC}-B curves is worth further theoretical investigation.

Equation (2) above has shown that both the second harmonic signal and the *d.c.* term carry the same γB term, indicating they have the same relationship with the applied external magnetic field B. This also explains the high consistency between $R^{2\omega}$-B and V_{DC}-B curves observed in our experiments (main text Fig. 4, Supplementary Figures 3, 4, 5, 8). Essentially, from the viewpoint of the vortex motion, both the second harmonic signal and the *d.c.* response come from the vortex motion in ratchet pinning potentials in 2D $NbSe_2$ crystals at $T \leq T_C$. According to the recent theory, in non-centrosymmetric superconductors, when vortices driven by the external charge current move among the asymmetric pinning potentials in the vortex flow regime, nonreciprocal charge transport will emerge[2]. The ratchet pinning potentials in few-layer $NbSe_2$ naturally come from disorder[2] like defects in 2D crystal[12,13] without inversion symmetry. Utilizing the extreme sensitivity of $NbSe_2$ in the superconducting regime, we can apply radio-frequency *a.c.* signals on the resistor fabricated on the same substrate, then the electromagnetic wave will radiate onto the $NbSe_2$ device nearby. The vortices in the 2D $NbSe_2$ will sense the *a.c.* oscillatory driving force and encounter a counterforce with different magnitudes for each direction of it. Consequently, the

vortex under such circumstances will acquire a net velocity and generate *d.c.* voltage[17,18]. Since both the second harmonic signal and the *d.c.* response occur in the vortex flow regime, we can also define phases based on the V_{DC}-B isotherms as shown in Supplementary Fig. 3. The absence of the vortex glass state in trilayer $NbSe_2$ at T = 2 K is due to its low melting temperature compared with the thicker (five-layer) sample[1]. Also, in the main text Figure 2, we calculated the maximum value of $\gamma' = 3.43\times10^4$ $T^{-1}A^{-1}$, which is larger than γ value 6.53×10^2 $T^{-1}A^{-1}$. Since γ value reflects the nonreciprocity of the system. This also explains the larger variation of V_{MAX2} than V_{MAX1} upon elevated a.c. power observed in our experiment.

Supplementary Note 3. Details about the direction of the electrical contacts, measurement configuration and possible heating effect.

During the experiments, we find that most of the cleavage edges of exfoliated $NbSe_2$ form a 60° angle (as shown in Supplementary Figure 4a). According to the previous study[19], theses cleavage edges correspond to zigzag edges of the crystal (as shown in Supplementary Figure 4b). Also, theory[2] has pointed out that for 2H-$NbSe_2$ with the trigonal crystal structure, the longitudinal nonreciprocal response ($V^{2\omega}$, the purple arrow in Supplementary Figure 4b) is maximum when the applied current is parallel to the zigzag direction of the crystal (an out-of-plane magnetic field is needed, as shown in Supplementary Figure 4b). So we intentionally fabricated electrical contacts along the cleavage edge of the $NbSe_2$ crystal, *i.e.*, the zigzag direction in the up-right panel of Supplementary Figure 4a. It should be noted that for 2D $NbSe_2$, the nonreciprocal charge transport occurs when vortices driven by the external charge current move among the asymmetric pinning potentials in the vortex flow regime. Here the vortices move in the in-plane direction and the magnetic field is in the out-of-plane direction. $NbSe_2$ doesn't have polarization in its crystal[2,3,12]. Note this is different from polar systems like $LaAlO_3/SrTiO_3$ oxide interface[20] and BiTeBr[21], where the nonreciprocal response is maximum when the polarization of the system (out-of-plane direction) and magnetic field (in-plane direction) and current are all orthogonal to each other.

Supplementary Note 4. Calculation and discussion on the responsivity of the 2D $NbSe_2$ antenna.

We calculate the responsivity of nonreciprocal charge transport in 2D $NbSe_2$, $\mathcal{R} = \frac{V_{Max}^{2\omega}}{I^2R}$. Taking the data from the main text Figure 4 at T = 5.25 K ($V_{Max}^{2\omega}$ = 1.775×10^{-6} V, excitation current $I = 2.5\times10^{-5}$ A and resistance R = 0.9 Ω), we obtain a responsivity $\mathcal{R}$ = 3.156×10^3 V/W. This value is indeed smaller than the state-of-the-art diodes which have the responsivity $\mathcal{R} \sim 10^4$ V/W. But the 2D $NbSe_2$ antenna device has its own unique advantages. (1) 2D $NbSe_2$ antenna can work at a temperature lower than 6.5K and control the motion of magnetic flux quanta, which has potential applications in quantum computing circuits.[18,19] (2) The physical origin of the rectifying effect between

the 2D $NbSe_2$ antenna and the commercial diode is completely different. The typical commercial diodes in the electronics community utilize the built-in electric field in semiconductor junctions to set the direction of the d.c. current, which don't have reversible nonreciprocal d.c. response and nonreciprocal multi-states. The 2D $NbSe_2$ antenna devices, however, utilize the principle of the intrinsic inversion symmetry breaking in the quantum crystals. This results in a reversible nonreciprocal d.c. response and nonreciprocal multi-states. These performances have unique potential applications in radio-frequency information storage and identification. (3) The 2D $NbSe_2$ antenna is an atomically-thin nano-scale device, which is far smaller than the commercial diodes (typically in sub-millimeter-scale). The nano-scale device can be used in flexible and transparent electrical circuit[22,23]. (4) Because the 2D $NbSe_2$ antenna utilizes the intrinsic inversion symmetry breaking of the crystal, the whole area of the sample is active. And the large active area is greatly demanded in electromagnetic wave harvesting (*e.g.* wireless charging or energy harvesting). For 2D $NbSe_2$, a large active area can be achieved by either integration of devices or fabrication of large homogenous samples. (5) The commercial diodes in the electronics community are very mature, and their performance has limited space to be improved, particularly owing to the built-in electric field of these Schottky diodes. Their rectification requires a threshold input voltage V_T = k_BT/e, known as thermal voltage (~26 meV at room temperature). This makes it challenging for the commercial diodes to harvest low-power (< 1 μW) electromagnetic waves[24], which is becoming increasingly crucial due to the fast-developing microwatts and nanowatts electronics. Also, their responsivity of the commercial diodes is limited by the transition time in diodes (typically orders of nanoseconds) and drops at high frequencies. Furthermore, there is a so-called terahertz gap (0.1 to 10 THz) of these commercial diodes that cannot be avoided[25]. Thus, low-power energy harvesters and terahertz detection are in great demand now. Scientists are searching for new materials and new rectification mechanisms for years. While the 2D $NbSe_2$ antenna devices utilize the intrinsic inversion symmetry breaking and work at a superconducting regime, they could be more efficient for harvesting low-power (<1 μW) electromagnetic waves. Also, the theory has predicted that 2D-TMDs can have an efficient second-order response even at ~THz scale[25]. We recognize that our 2D $NbSe_2$ antenna device doesn't reach that high frequency for now. However, we are the first to push 2D single-crystalline superconductors into practical device applications. Note that the frequency at 900 MHz is much larger than the resonant frequency of the vortex moving in the artificial periodic potential (~ 100 MHz)[11]. Note that for a nonreciprocal change transport in an asymmetric pinning potential, the upper frequency of the vortex must be smaller than the upper-frequency limit (resonate frequency) in order to allow the vortex move longer than the unit-cell distance, typically at a scale of ~μm for artificial fabricated asymmetric pinning potentials in one a.c. cycle[17,18]. In 2D $NbSe_2$, however, the point defects in the crystal which form the asymmetric pinning potentials are regulated by the crystal lattice. Thus, the unit-cell distance of asymmetric pinning potentials in 2D $NbSe_2$ should be corresponding to its own lattice constant, typically at a scale of ~nm. For comparison, the resonant frequency limit of the vortex moving in the artificial periodic potential is limited to only about 100 MHz[11]. We can thus estimate

that the upper-frequency limit of our 2D $NbSe_2$ should be ~ 10 GHz or even larger[25]. This explains the significantly higher sensing frequency of 900 MHz in our 2D $NbSe_2$ antenna device, suggests that it great potential to realize sensitive detection at much higher frequencies in the future. Here, we do not intend to propose an immediate commercial application. But the new mechanism and new device functionalities demonstrated in this work suggest that 2D single-crystalline superconductors are great candidates for the building blocks of next-generation wireless networks that may address some of the major limitations of the current technologies with proper device optimizations[24].

Supplementary Note 5. Discussion about the response frequency of the $NbSe_2$ antenna device.

For the device in the main text Figure 5, the device shows a prominent d.c response at $f_{IN} \geq$ 5MHz. Note at 1MHz < f_{IN} < 5MHz, the d.c. response is not so prominent (Supplementary Figure 9d). We further measure the d.c. response of this device via monitoring the dynamic behavior of the device with the *a.c.* signal (Supplementary Figure 9e), which shows that the device also exhibits the d.c response at f_{IN} = 1 MHz. Considering all the atomically thin device we measured, most devices shows the response at $f_{IN} \geq$ 1 MHz.

Supplementary Note 6. The discussion of the possible heating effect.

As shown in Supplementary Figure 8a, as we increase the applied a.c. signal power, the spectrum characteristic of the d.c. response doesn't change. Also, the vortex flow regime of the $NbSe_2$ antenna device doesn't vanish during the application of the a.c. signal as shown in the main text Figure 4e and Figure 5c, indicative of the stable vortex dynamic state without quenching to the normal state (by the heating effect). Noted that in the main text Figure 5c, as we increase the applied power, the peak position shrinks a little bit. This is because a larger a.c. signal power would drive more vortices to move among the asymmetric pinning potentials in $NbSe_2$ crystals and enhance their moving speed. As a result, a larger resistance would appear under the same magnetic field and the vortex flow regime of the device would shrink[1,11,26]. As shown in the main text Figure 5 c-e (also see Supplementary Figure 8 a-c), as we increase the applied a.c. signal power, the d.c response the device of the device is also increased, this means that the shrink of the peak position is not coming from the heading effect because the increase of temperature would reduce the d.c. response (Supplementary Figure 9 a-c). We also do the control experiment by separating the resistor and the $NbSe_2$ channel. As schematically shown in Supplementary Figure 10a, the resistor and the $NbSe_2$ channel are fabricated on two different SiO_2/Si wafers, there is a gap between these two wafers which could almost block out the heating effect of the applied a.c. signal. As we increase the power applied on the resistor, the d.c. response increases and the peak positions also shift. Note that this behavior is consistent with the main text Figure 5c, which also proves our argument that the shift of the peak position is not coming from

the heating effect. We do realize that the heating effect in our device is unavoidable. However, based on the aforementioned reasons we believe that the heating effect on the nonreciprocal d.c. sensitivity is not significant and the dynamical phase diagram of $NbSe_2$ is not qualitatively altered.

Supplementary Note 7.Thickness-dependent antisymmetric second harmonic and *d.c.* response in $NbSe_2$.

Combining the results in Supplementary Figures 6-9, 11-15 and the main text, we find that the antisymmetric second harmonic and the *d.c.* response can be detected in few-layer $NbSe_2$ while not in bulk $NbSe_2$. We attribute this to the weak interlayer coupling in $NbSe_2$, which agrees with the Ising superconductivity and the observed unusual continuous paramagnetic-limited phase transition[27] due to inversion symmetry breaking. As the sample thickness increases, the interlayer coupling becomes more important by introducing orbital effects[28]. However, the orbital effect is significantly suppressed when the sample thickness is smaller than the out-of-plane penetration depth[16] (~23 nm; ref. [29]). Considering the γ value proposed by 2H-type TMD systems[3]:

$$\gamma = \frac{4\pi m\Lambda}{We\hbar k_B T_c},$$

where W is the width of the device channel, and

$$\Lambda \sim \frac{g\mu_B \Delta_{SO}\Gamma}{(\pi k_B T_c)^2}.$$

Here, Δ_{SO} is the spin splitting due to the spin-orbit interaction, g is the g-factor, Γ is the parameter of the trigonal warping[30] and μ_B is the Bohr magneton. From the above equation, we could find that Δ_{SO} is a very import parameter affecting the γ value[8,20]. We believe that the nonreciprocity still dominates the vortex motion as long as the interlayer coupling constant is smaller than the spin splitting energy Δ_{SO} which is satisfied in few-layer $NbSe_2$[28]. While in the bulk limit, the orbital effect will dominate, resulting in a zero second harmonic and *d.c.* response. We note that the analysis here is qualitative rather than quantitative. Further experimental and theoretical studies are needed to fully understand this.

Supplementary Note 8. Large enhancement of γ value in the superconducting regime In 2D $NbSe_2$.

According to proposed theories[2,3], the nonreciprocal paraconductivity in the superconducting fluctuation regime can be used to explain the enhanced γ value below the superconducting transition temperature. At superconducting fluctuation regime, the γ_S value (γ value below the superconducting transition temperature) for 2H-transition-metal dichalcogenide is[2]

$$\gamma_S \sim \frac{m\Delta_{SO}\lambda}{eT_0^3} \tag{5}$$

Here, e is the electron charge, m is the electron mass, Δ_{SO} is the spin-orbit splitting, λ is the trigonal warping parameter, and T_0 the mean-field superconducting transition temperature. While in the normal state, the γ_N value (γ value in the normal state) can be acquired using the Boltzmann equation, the typical value is calculated as[3]

$$\gamma_N \sim \frac{m\Delta_{SO}\lambda}{eE_F^3} \tag{6}$$

Here, E_F the Fermi energy. According to the BCS theory, the superconducting gap $\Delta \sim T_0$, then we can get[2]

$$\frac{\gamma_S}{\gamma_N} \sim \left(\frac{E_F}{T_0}\right)^3 \sim \left(\frac{E_F}{\Delta}\right)^3 \tag{7}$$

Equation (7) here shows that the ratio of γ_S/γ_N is proportional to the energy scale difference between the Fermi energy E_F (~100 meV) and the superconducting gap Δ(~1 meV). Thus we can estimate that γ_S is around 10^6 times of γ_N. This explains the large enhancement of γ value in the superconducting regime in 2D $NbSe_2$.

Supplementary References

1. Benyamini, A. *et al.* Fragility of the dissipationless state in clean two-dimensional superconductors. *Nat. Phys.* **15**, 947–953 (2019).
2. Hoshino, S., Wakatsuki, R., Hamamoto, K. & Nagaosa, N. Nonreciprocal charge transport in two-dimensional noncentrosymmetric superconductors. *Phys. Rev. B* **98**, (2018).
3. Wakatsuki, R. *et al.* Nonreciprocal charge transport in noncentrosymmetric superconductors. *Sci. Adv.* **3**, e1602390 (2017).
4. Lustikova, J. *et al.* Vortex rectenna powered by environmental fluctuations. *Nat. Commun.* **9**, 4922 (2018).
5. Pop, F., Auban-Senzier, P., Canadell, E., Rikken, G. L. J. A. & Avarvari, N. Electrical magnetochiral anisotropy in a bulk chiral molecular conductor. *Nat. Commun.* **5**, 3757 (2014).
6. Wakatsuki, R. & Nagaosa, N. Nonreciprocal current in noncentrosymmetric rashba superconductors. *Phys. Rev. Lett.* **121**, (2018).
7. Qin, F. *et al.* Superconductivity in a chiral nanotube. *Nat. Commun.* **8**, 14465 (2017).
8. Yasuda, K. *et al.* Nonreciprocal charge transport at topological insulator/superconductor interface. *Nat. Commun.* **10**, 2734 (2019).
9. Plourde, B. L. T. Nanostructured superconductors with asymmetric pinning potentials: vortex ratchets. *IEEE Trans. Appl. Supercond.* **19**, 3698–3714 (2009).
10. Giaever, I. Magnetic Coupling Between Two Adjacent Type-II Superconductors. *Phys. Rev. Lett.* **15**, 825–827 (1965).
11. Moshchalkov, V., Woerdenweber, R. & Lang, W. *Nanoscience and Engineering in Superconductivity*. (Springer Science & Business Media, 2010).
12. Itahashi, Y. M., Saito, Y., Ideue, T., Nojima, T. & Iwasa, Y. Quantum and classical ratchet motions of vortices in a two-dimensional trigonal superconductor. *Phys. Rev. Research* **2**, 023127 (2020).
13. Zhao, K. *et al.* Disorder-induced multifractal superconductivity in monolayer niobium dichalcogenides. *Nat. Phys.* **15**, 904–910 (2019).
14. Koshelev, A. E., Sadovskyy, I. A., Phillips, C. L. & Glatz, A. Optimization of vortex pinning by nanoparticles using simulations of the time-dependent Ginzburg-Landau model. *Phys. Rev. B* **93**, 060508 (2016).

15. Coffey, M. W. & Clem, J. R. Unified theory of effects of vortex pinning and flux creep upon the rf surface impedance of type-II superconductors. *Phys. Rev. Lett.* **67**, 386–389 (1991).
16. Tinkham, M. *Introduction to Superconductivity*. (Courier Corporation, 2004).
17. Villegas, J. E. A superconducting reversible rectifier that controls the motion of magnetic flux quanta. *Science* **302**, 1188–1191 (2003).
18. Silva, C. C. de S., Vondel, J. V. de, Morelle, M. & Moshchalkov, V. V. Controlled multiple reversals of a ratchet effect. *Nature* **440**, 651–654 (2006).
19. Guo, Y. *et al.* Distinctive in-Plane Cleavage Behaviors of Two-Dimensional Layered Materials. *ACS Nano* **10**, 8980–8988 (2016).
20. Choe, D. *et al.* Gate-tunable giant nonreciprocal charge transport in noncentrosymmetric oxide interfaces. *Nat. Commun.* **10**, 4510 (2019).
21. Ideue, T. *et al.* Bulk rectification effect in a polar semiconductor. *Nature Physics* **13**, 578–583 (2017).
22. Fiori, G. *et al.* Electronics based on two-dimensional materials. *Nat. Nanotechnol.* **9**, 768–779 (2014).
23. Novoselov, K. S., Mishchenko, A., Carvalho, A. & Neto, A. H. C. 2D materials and van der Waals heterostructures. *Science* **353**, aac9439 (2016).
24. Hemour, S. & Wu, K. Radio-frequency rectifier for electromagnetic energy harvesting: development path and future outlook. *Proc. IEEE* **102**, 1667–1691 (2014).
25. Isobe, H., Xu, S.-Y. & Fu, L. High-frequency rectification via chiral Bloch electrons. *Sci. Adv.* **6**, eaay2497 (2020).
26. Anderson, P. W. & Kim, Y. B. Hard superconductivity: theory of the motion of Abrikosov flux lines. *Rev. Mod. Phys.* **36**, 39–43 (1964).
27. Sohn, E. *et al.* An unusual continuous paramagnetic-limited superconducting phase transition in 2D $NbSe_2$. *Nat. Mater.* **17**, 504–508 (2018).
28. Xi, X. *et al.* Ising pairing in superconducting $NbSe_2$ atomic layers. *Nat. Phys.* **12**, 139–143 (2016).
29. de Trey, P., Gygax, S. & Jan, J.-P. Anisotropy of the Ginzburg-Landau parameter κ in $NbSe_2$. *J. Low Temp. Phys.* **11**, 421–434 (1973).
30. Kormányos, A. *et al.* Monolayer MoS_2: Trigonal warping, the Γ valley, and spin-orbit coupling effects. *Phys. Rev. B* **88**, 045416 (2013).
